# Structural modification and antibacterial property studies of natural chalcone sanjuanolide

**DOI:** 10.3389/fchem.2022.959250

**Published:** 2022-08-05

**Authors:** Jiadai Zhai, Shucheng Li, Lin Fu, Chuang Li, Bingxia Sun, Feng Sang, Hongliang Liu

**Affiliations:** ^1^ School of Pharmaceutical Science and Technology, Tianjin University, Tianjin, China; ^2^ School of Life Sciences and Medicine, Shandong University of Technology, Zibo, China

**Keywords:** chalcone, hydroxyisoprenyl moiety, derivatives, synthesis, antibacterial activity

## Abstract

Clinical infections arise from multidrug-resistant bacteria and pose a serious threat to human and global public health. Moreover, due to very few antibiotics being discovered, there is an urgent need to develop new antibacterial agents to combat antimicrobial resistance challenges. In this study, a series of new chalcone derivatives bearing a 3′-hydroxyisoprenyl moiety were prepared to employ Claisen–Schmidt condensation as a key step by combinatorial chemistry, and overall yields of these novel derivatives are in the range of 28–68% in the two-step reaction. Sanjuanolide and the synthesized derivatives have been investigated for their expected antibacterial activities against Gram-positive bacteria (*Staphylococcus aureus* CMCC 26003) and Gram-negative bacteria (*Escherichia coli* CMCC 44102). Among these compounds, only **4c** (MIC = 12.5 μg/ml) and **4d** (MIC = 25 μg/ml) exhibited antibacterial activity comparable to sanjuanolide (MIC = 12.5 μg/ml, against *S. aureus* CMCC 26003), and the results of subsequent *in vivo* experiments on sanjuanolide suggest that sanjuanolide exhibits bacteriostatic and bactericidal effects by altering the cellular structure, disrupting the integrity of cell membranes, and reducing the outer membrane potential.

## 1 Introduction

Chalcones, which contain a three-carbon α, β-unsaturated carbonyl system, have been reported to have therapeutic potential, including antibacterial (Nielsen et al., 2005; Chen et al., 2010; Zhuang., 2017; Duvauchelle et al., 2021) and anticancer activities (Kamal et al., 2010; Salehi et al., 2021). Among the numerous chalcones and their derivatives, a class of chalcones bearing the 3′-hydroxyisoprenyl moiety had received less attention until sanjuanolide was isolated by Shaffer et al. (2016). Considering the slightly greater cytotoxic activities of sanjuanolide, we completed the first synthesis and preliminary structure–activity relationship of sanjuanolide and its eight analogs (Zhai et al., 2019). Then, the total synthesis and antibacterial activities of natural chalcones bearing the 3′-hydroxyisoprenyl (Li et al., 2019) moiety were also investigated in our laboratory.

**FIGURE 1:**
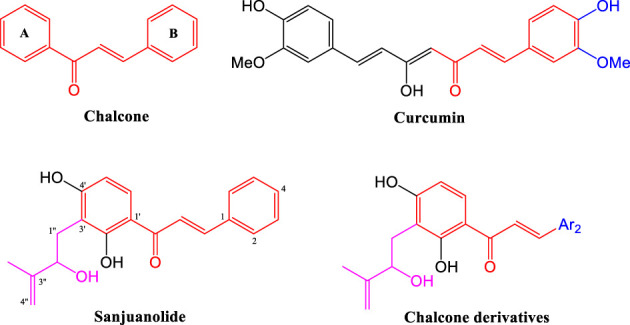
Structures of chalcone, curcumin, sanjuanolide, and their new derivatives.

Our previous studies suggest that the hydroxyisoprenyl moiety has a positive effect on their antibacterial activity (Li et al., 2019). As the basic structural unit of many biological active natural products, heterocyclic aromatic (Zheng et al., 2011; Lagu et al., 2020) and phenolic hydroxyl (Yang et al., 2007; Ammaji et al., 2022) derivatives have been used in the structural modification of chalcones several times, and the existence of these special units in chalcone derivatives is critical (Ohkuma et al., 2008; Kasetti et al., 2021). Therefore, these characteristic structural units will provide an important reference to the design of active derivatives. In this work, sanjuanolide and curcumin were used as reference compounds (shown in Figure 1). We designed and synthesized a new series of chalcone derivatives, in which the 3′-hydroxyisoprenyl moiety was reserved, and the benzene ring (B-ring) was replaced with heterocyclic aromatic (pyridine, thiofuran, furan, and pyrrole) or phenolic derivatives (di-substituted, the same as curcumin). A total of 19 chalcone derivatives bearing the 3′-hydroxyisoprenyl moiety were designed, but only ten derivatives were successfully synthesized and then evaluated for their antibacterial activity against Gram-positive bacteria (*Staphylococcus aureus* CMCC 26003) and Gram-negative bacteria (*Escherichia coli* CMCC 44102). Sanjuanolide was used as a positive control and selected for subsequent bacteriostatic analysis and flow cytometry analysis with regard to the antibacterial effects and properties.

## 2 Results and discussion

### 2.1 Synthesis

**FIGURE 2:**
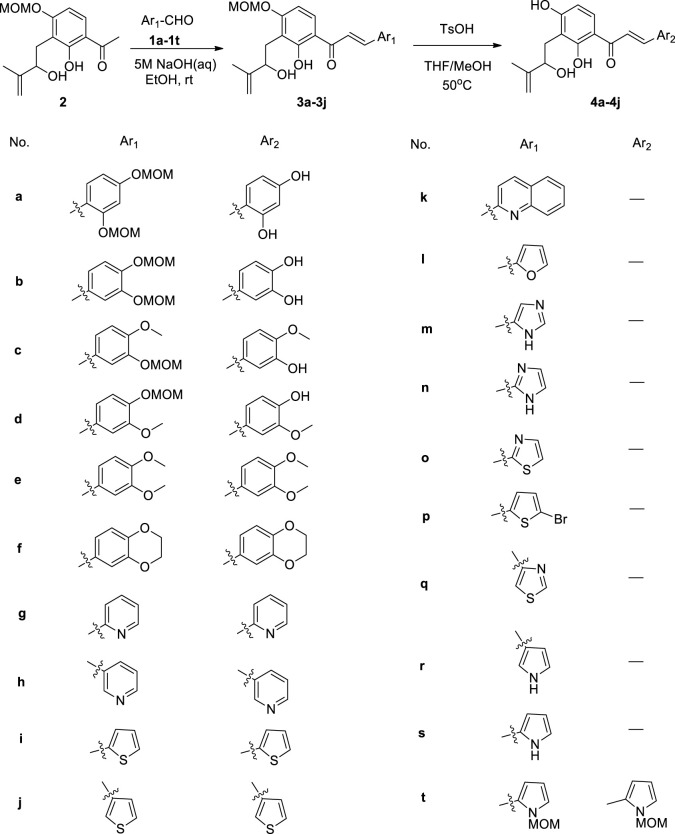
Synthesis of new chalcone derivatives **4a**–**4j**.

First, we have prepared the intermediate **2** by Schenck-ene reaction, as previously mentioned (Li et al., 2019). Subjecting compound **2** to Claisen–Schmidt condensation with various aldehydes (**1a**–**1t**) (Effenberger et al., 1997; Jogireddy et al., 2006; Liegault et al., 2009; Kim et al., 2014; Hotsumi et al., 2019), using 5M NaOH as the base, afforded adduct products (**3a**–**3j**, **3t**). Then, deprotection of **3a**–**3j** by using TsOH to obtain target compounds **4a**–**4j** and overall yields of **4a**–**4j** are in the range of 28–68% in the two-step reaction. All the target compounds were characterized by ^1^H NMR, ^13^C NMR, and high-resolution mass spectrometry (HRMS). In addition, nine other heterocyclic aldehydes (**1k**–**1s**) were also subjected to Claisen–Schmidt condensation, but no conversion could be observed in most cases. Although the MOM-protected pyrrole aldehyde (**1t**) can be reacted with compound **2**, the MOM group remains unchanged in the subsequent deprotection section (shown in Figure 2).

### 2.2 Biological evaluation

#### 2.2.1 Antibacterial activity *in vitro*

*In vitro* antibacterial activities of sanjuanolide, curcumin, and the synthesized derivatives **(4a–4j)** are shown in Table 1. The results demonstrate that **4c** (MIC = 12.5 μg/ml) and **4d** (MIC = 25 μg/ml) exhibited stronger inhibitory activity against *S. aureus* CMCC 26003 than any other new derivatives. Both **4c** (3-hydroxyl, 4-methoxy) and **4d** (3-methoxy, 4-hydroxyl) contain one hydroxyl and one methoxy substitution, which is similar to the structure of the reference compound curcumin, suggesting that this combination (3-hydroxyl, 4-methoxy or 3-methoxy, and 4-hydroxyl) may be more conducive to the maintenance of the antibacterial activity against *S. aureus* CMCC 26003. On the other hand, when the C-3 and C-4 positions were substituted by dihydroxyl (**4b**, MIC = 100 μg/ml) or dimethoxy (**4e**, MIC >200 μg/ml), the antibacterial activity of the chalcone derivatives decreased significantly, and the same effect also occurred in **4a** and **4f**. Moreover, heterocyclic-modified chalcone derivatives (**4g**–**4j**) did not exhibit observable antibacterial activity against *S. aureus* CMCC 26003 (MIC >200 μg/ml). In addition, all the chalcone derivatives are devoid of obvious inhibitory activity against *E*. *coli* CMCC 44102 at the highest test concentration (200 μg/ml), and curcumin did not show any inhibitory activity against the two test strains.

**TABLE 1:** Minimal inhibitory concentration (MIC) of curcumin, sanjuanolide, and its new derivatives (**4a**–**4j**).

Compound	*S. aureus* CMCC 26003	*E*. *coli* CMCC 44102
Sanjuanolide	12.5	>200
Curcumin	>200	>200
**4a**	>200	>200
**4b**	100	>200
**4c**	12.5	>200
**4d**	25	>200
**4e**	>200	>200
**4f**	>200	>200
**4g**	>200	>200
**4h**	>200	>200
**4i**	>200	>200
**4j**	>200	>200

*MIC (in μg/mL) values. Assay experiments were performed in duplicates at three independent times.

**FIGURE 3:**
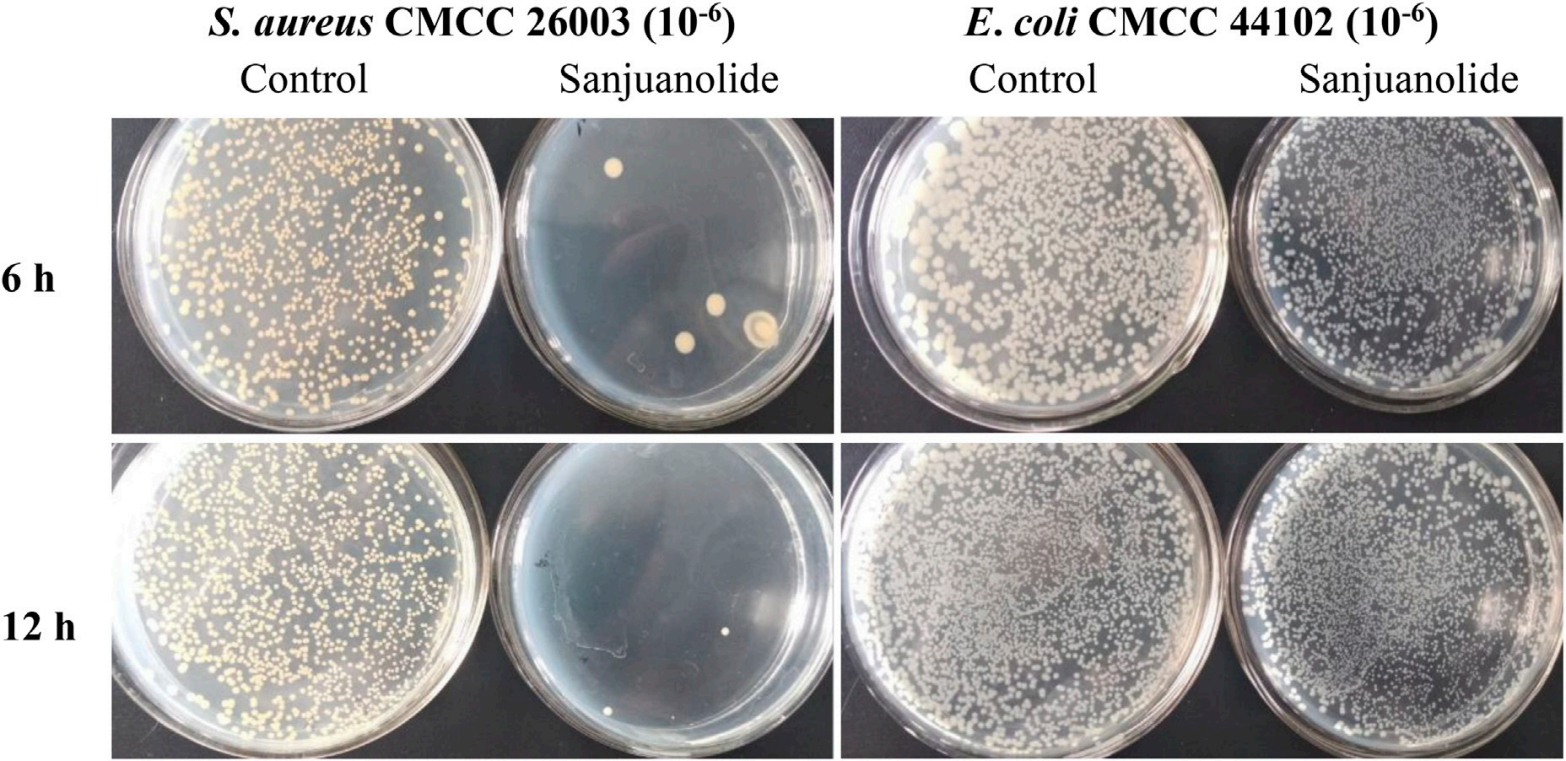
Antibacterial effect of sanjuanolide determined using the plate counting assay.

After the test, strains were incubated for 12 h, and *S. aureus* CMCC 26003 and *E*. *coli* CMCC 44102 were tenfold gradiently diluted to 10^−6^ and inoculated on Mueller–Hinton agar (MHA) medium containing 12.5 μg/ml of sanjuanolide for 6 and 12 h. Sanjuanolide showed a complete inhibitory activity against *S. aureus* CMCC 26003, whereas *E*. *coli* CMCC 44102 showed total drug resistance to sanjuanolide (Figure 3).

#### 2.2.2 Effect of sanjuanolide on cell morphology

**FIGURE 4:**
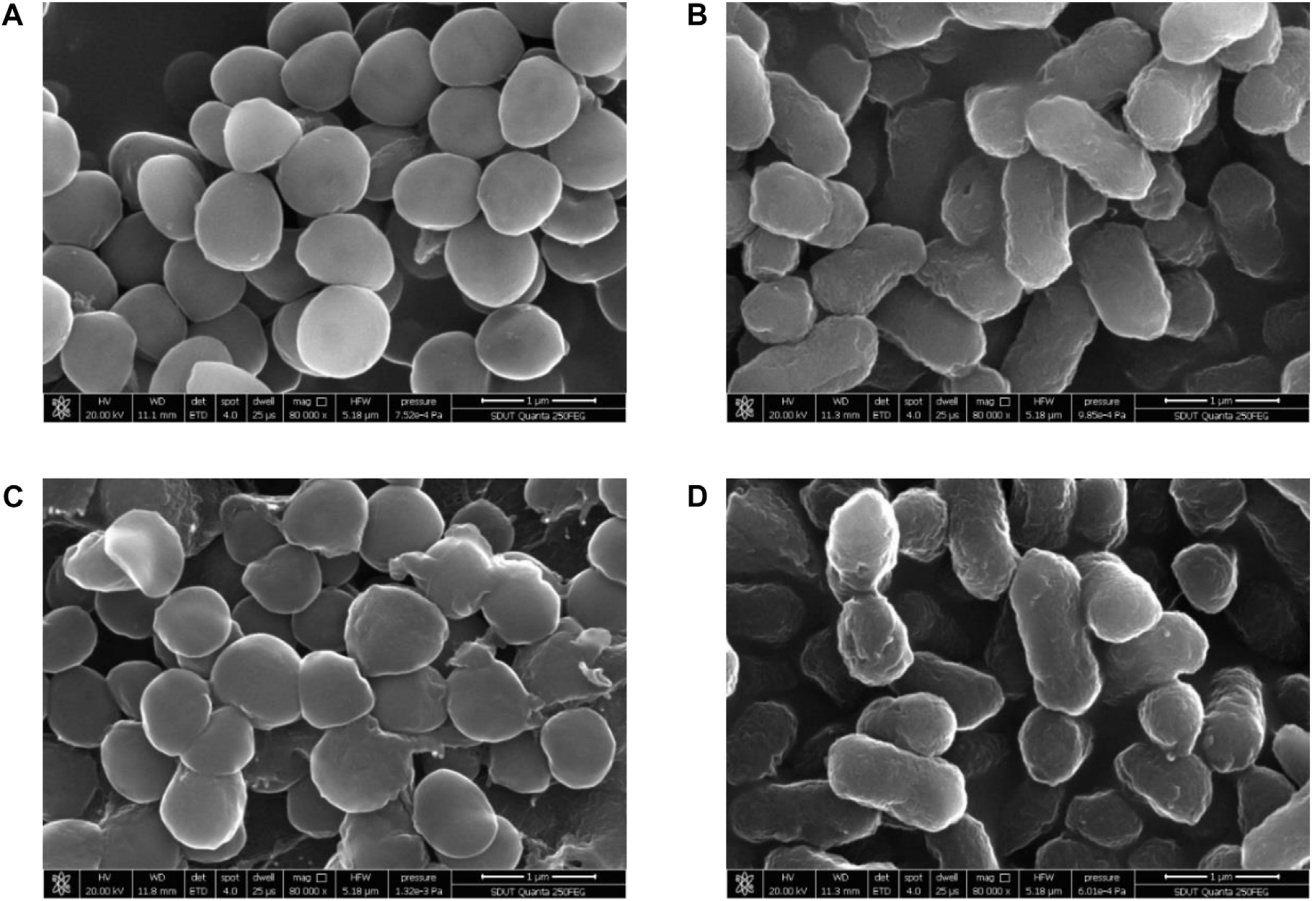
SEM images of *S. aureus* CMCC 26003 and *E*. *coli* CMCC 44102 in the absence of sanjuanolide **(A,B)** and presence of sanjuanolide **(C,D)**.

The changes in the cellular surface microstructure of *S. aureus* CMCC 26003 and *E*. *coli* CMCC 44102 under the stress treatment of sanjuanolide were studied using scanning electron microscopy **(**SEM) analysis. In the control groups, the cellular surface microstructures of *S. aureus* and *E. coli* CMCC 44102 were intact and uniform in size (Figures 4A, B). The cellular surface microstructure of *S. aureus* showed significant differences under the stress treatment of sanjuanolide, which appeared to be severely wrinkled, collapsed, and even broken (Figure 4C). In contrast, in the presence of sanjuanolide, the *E*. *coli* CMCC 44102 cells showed slight changes (Figure 4D). These results suggest that sanjuanolide may cause severe cellular structure damage to *S. aureus*, which results in cell death. The SEM analyses were consistent with the results of the antibacterial tests.

#### 2.2.3 Effect of sanjuanolide on membrane permeability

**FIGURE 5:**
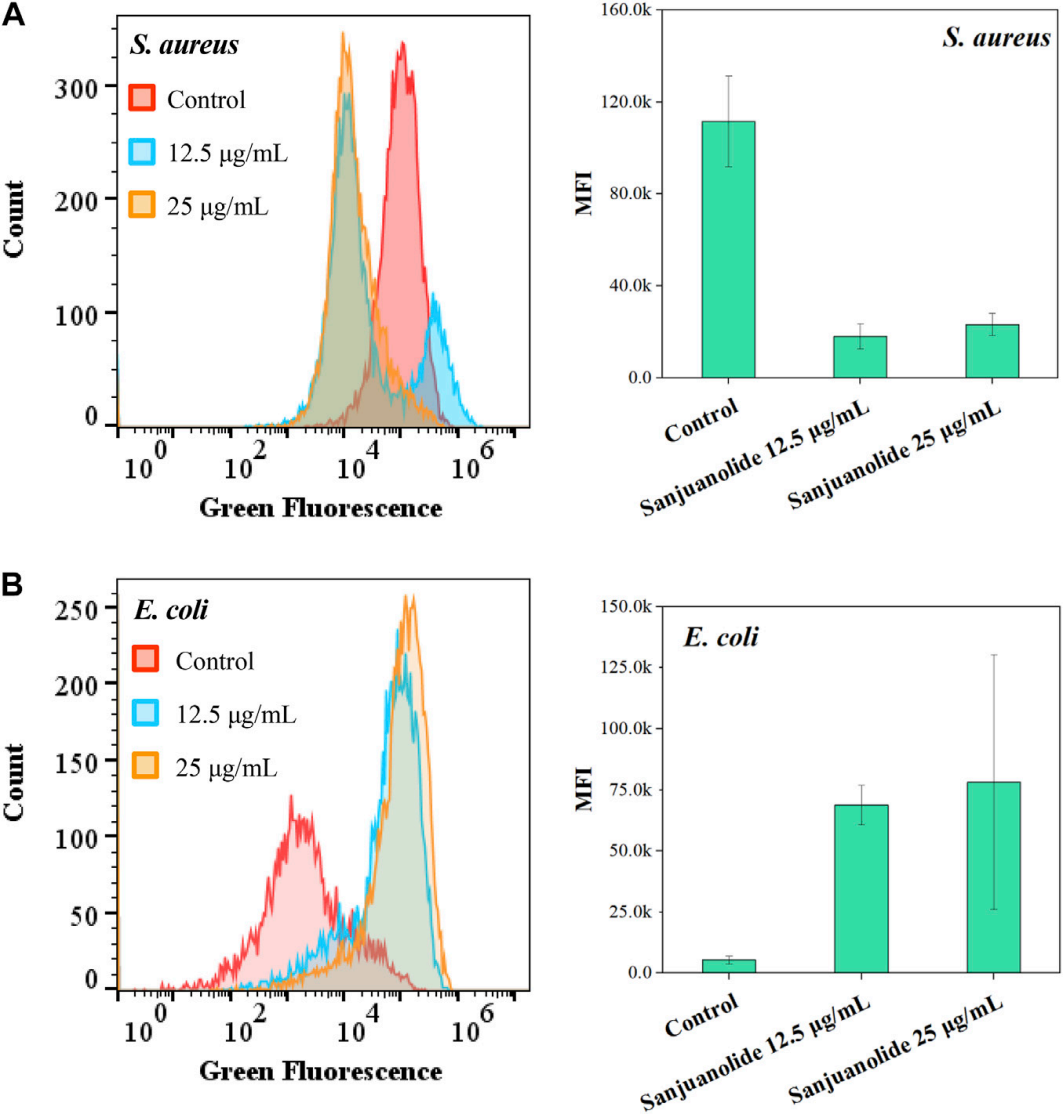
Fluorescence histogram superimposition and mean fluorescence intensity (MFI) of *S. aureus* CMCC 26003 **(A)** and *E. coli* CMCC 44102 **(B)** exposed to different concentrations of sanjuanolide.

5(6)-Carboxyfluorescein diacetate (cFDA) was a non-fluorescent precursor that can be taken up by living cells, and the diacetate groups were hydrolyzed intracellularly by non-specific esterases to produce a highly fluorescent carboxyfluorescein (cF) (Hoefel et al., 2003). As shown in Figure 5, sanjuanolide induced entirely different results for cF fluorescence intensity in *S. aureus* CMCC 26003 and *E*. *coli* CMCC 44102. When *S. aureus* CMCC 26003 was stressed by 12.5 and 25 μg/ml of sanjuanolide, the fluorescence intensity of cF showed a substantial left shift, indicating a gradient decrease in the fluorescence intensity, and 25 μg/ml of Sanjuanolide showed greater influence on the fluorescence intensity (Figure 5A). In contrast, with regard to *E. coli* CMCC 44102, the fluorescence intensity gradually increased along with the increased concentration of sanjuanolide (Figure 5B). cFDA, being hydrophobic, can diffuse into the cell across the phospholipid bilayer, whereas cF, being hydrophilic, is hindered from diffusing out of the cell. In contrast, the outer membrane of Gram-negative bacteria is highly impermeable to hydrophobic molecules (Vaara, 1992). Indeed, this explains the strong resistance of these bacteria to hydrophobic antibiotics, detergents, and dyes. cFDA is hydrophobic and can diffuse into the cell through the phospholipid bilayer, while cF is hydrophilic and will hinder diffusion out of the cells (Morono et al., 2004). Although the addition of EDTA increased the permeability of the outer membrane to hydrophobic solutes, it did not promote the leakage of cF from the cell into the reaction solution, which caused the phenomenon of the higher fluorescence intensity of *E. coli* CMCC 44102. This result indicated that sanjuanolide did not affect the membrane integrity of *E. coli* CMCC 44102, and cF was detained within the cell, whereas the decrease in the fluorescence intensity of *S. aureus* CMCC 26003 was due to the outflow of cF caused by the cell membrane breakage.

All the aforementioned pieces of evidence suggest that sanjuanolide has an intense effect on the cell membrane permeability of Gram-positive *S. aureus* CMCC 26003, with increasing concentrations causing increasing damage to the cell membrane and subsequent apoptosis, while its effect on *E. coli* CMCC 44102 is inconspicuous.

#### 2.2.4 Effect of sanjuanolide on membrane potential

DiOC_2_(3) emits green fluorescence in all bacterial cells, and the higher membrane potential causes the dye molecules to self-polymerize, which increases the red fluorescence signal ratio (Novo et al., 1999; Novo et al., 2000). This causes the fluorescence of the dye to migrate toward the red emission wavelength, which increases the red fluorescence intensity. Proton ion carriers, such as CCCP, disrupt the membrane potential by eliminating the proton gradient, resulting in a decrease in the red fluorescence intensity and a decrease in the red/green fluorescence signal ratio. The extent of membrane damage is assessed by calculating the red/green ratio of the integrated fluorescence, which is derived from the MFI (Figure 5).

**FIGURE 6:**
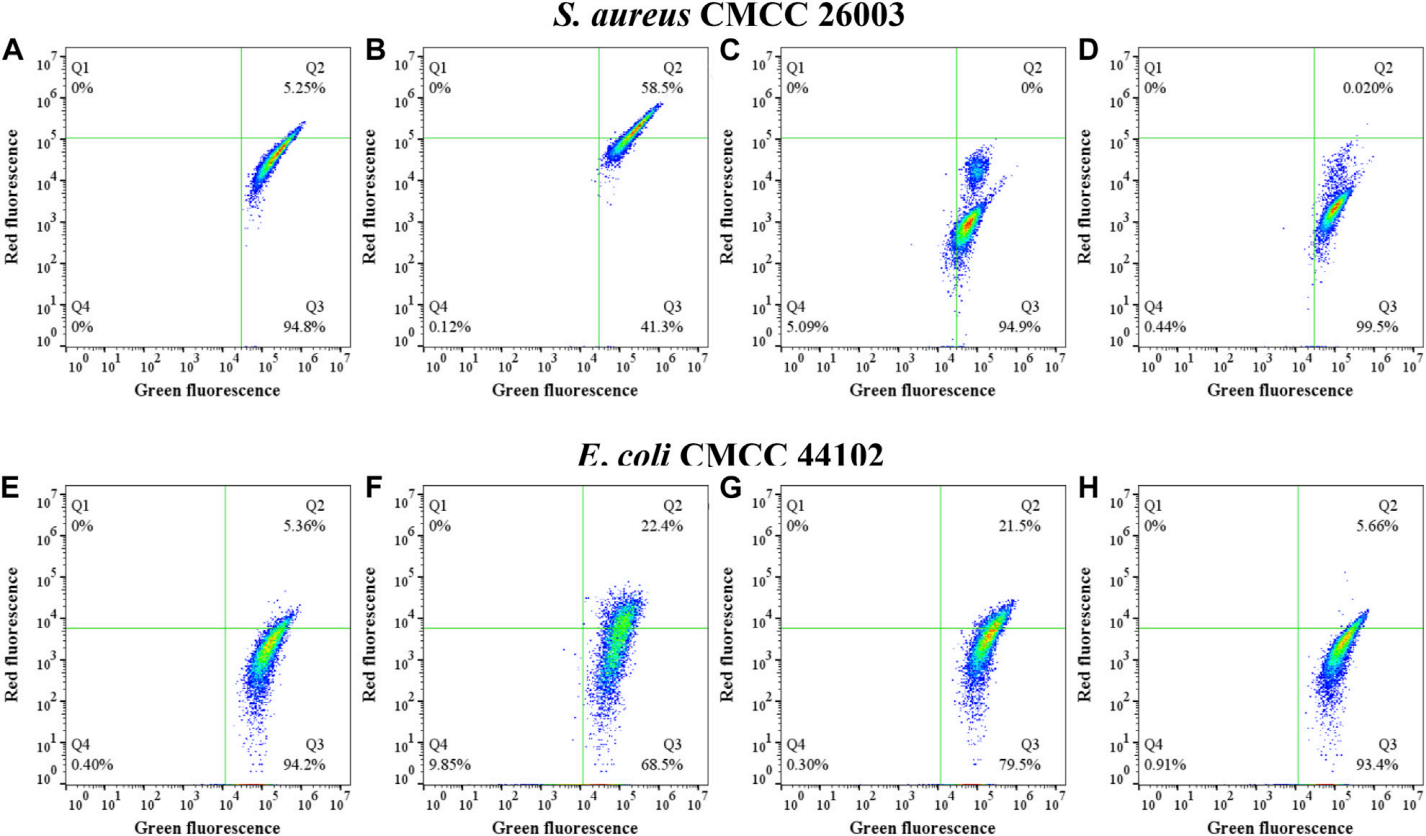
Measurement of the bacterial membrane potential of different treatments of *S. aureus* CMCC 26003 **(A–D)** and *E. coli* CMCC 44102 **(E–H)** as determined by flow cytometry. **(A/E)** CCCP+ and **(B/F)** CCCP−, **(C/G)** with 12.5 μg/ml sanjuanolide **(D/H)** and 25 μg/ml sanjuanolide.

According to the results of flow cytometric analysis in the CCCP+ group, approximately 95% of the cells (Q3 region) were stained with green fluorescence after depolarization treatment of *S. aureus* CMCC 26003 samples (Figure 6A). Compared to the negative group (Figure 6B CCCP− group), treatment with sanjuanolide decreased the number of bacteria in the Q2 region (Figures 6C, D), indicating that sanjuanolide can depolarize the cells and disrupt the cell membrane potential, as in the positive group with the addition of CCCP. Moreover, the superimposed plots also clearly exhibited this trend (Figure 6); when the CCCP− group was supplemented by sanjuanolide with concentrations up to 12.5 and 25 μg/ml, the cells showed a substantial decrease in red fluorescence, indicating the depolarization of the bacteria.

**FIGURE 7:**
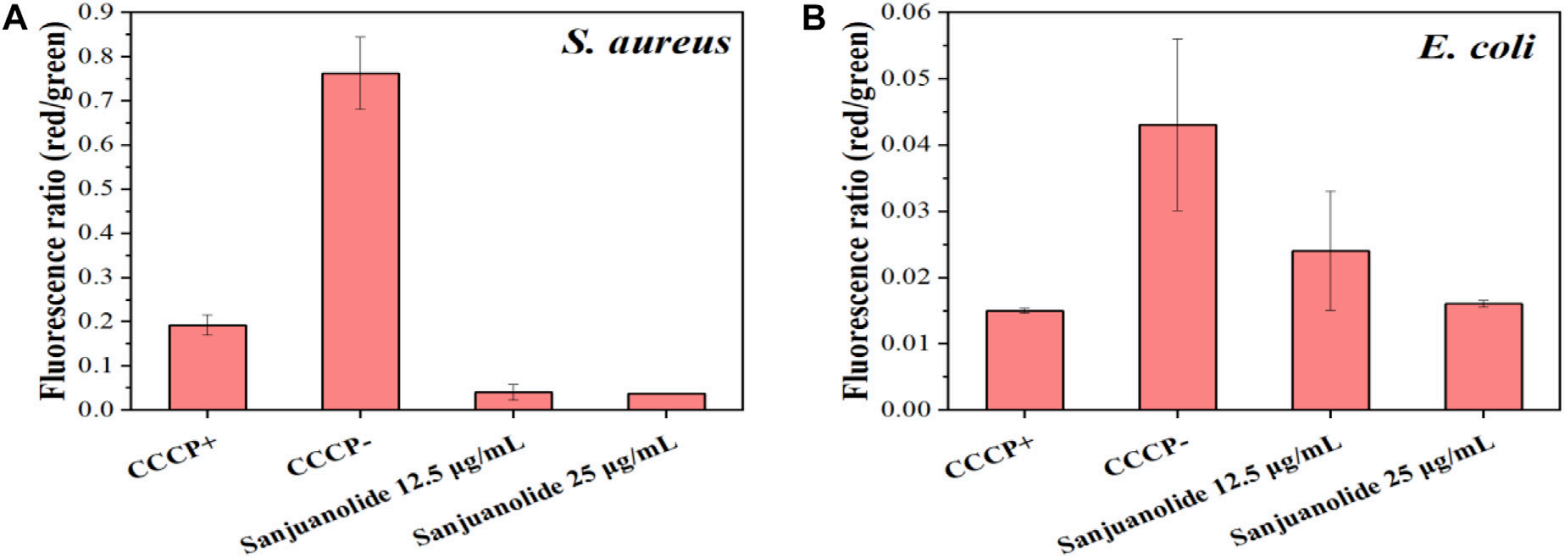
Ratio of red/green fluorescence intensities of *S. aureus* CMCC 26003 **(A)** and *E*. *coli* CMCC 44102 **(B)** under the different treatments, which were calculated using the MIF.

The lower red-green ratio in the statistical plot also indicated that sanjuanolide could inhibit or kill bacteria by disrupting their membrane potential (Figure 7A), and the addition of sanjuanolide decreased the red/green ratio from 0.762 in the negative control to a level similar to CCCP+ and continuously decreased with increasing sanjuanolide concentration. Compared to *S. aureus* CMCC 26003, where there was a clear trend, the change in the membrane potential of *E. coli* CMCC 44102 after sanjuanolide treatment was not as pronounced (Figure 7B), and its membrane potential also tended to depolarize with the increasing natural product concentration as well but probably did not affect the growth state of *E. coli* CMCC 44102, as shown in Figure 3 and Figure 6 (**E–H**). Similarly, Figure 6B showed the same trend with the negative group as the control. Therefore, sanjuanolide may lead to membrane rupture and leakage of contents by disrupting the membrane potential of bacteria, ultimately leading to bacterial death, and this effect was particularly pronounced in *S. aureus* CMCC 26003.

## 3 Experiment

### 3.1 General

Reagents purchased from commercial suppliers were used without purification, unless otherwise noted. All reactions were carried out under an argon atmosphere, unless otherwise noted. Solvents for chromatography were used as supplied by Jinan Fusheng (Shandong, China). Reactions were monitored by thin layer chromatography (TLC) carried out on silica gel plates using UV light as a visualizing agent. Then, 200–300 mesh silica gel purchased from Yantai Jiangyou (Shandong, China) was used for column chromatography. ^1^H NMR and ^13^C NMR were recorded on a Bruker Avance III 400 apparatus and calibrated by using internal references and solvent signals CDCl_3_ (*δ*_H_ = 7.26 ppm; *δ*_C_ = 77.16 ppm), acetone-*d*_6_ (*δ*_H_ = 2.05 ppm; *δ*_C_ = 206.68, 29.92 ppm), DMSO-*d*_6_ (*δ*_H_ = 2.50 ppm; *δ*_C_ = 39.52 ppm), and CD_3_OD (*δ*_H_ = 3.34 ppm; *δ*_C_ = 49.00 ppm). ^1^H NMR data are reported as follows: chemical shift, multiplicity (s = singlet, d = doublet, t = triplet, q = quartet, br = broad, and m = multiplet), coupling constants, and integration. IR spectra were recorded on a Nicolet5700 instrument. High-resolution mass spectra (HRMS) were detected by using a Varian 7.0T FTMS instrument.

cFDA (≥90.0% (HPLC)) and 3,3′-diethyloxacarbocyanine iodide (DiOC_2_ (3), 98%) were purchased from Aladdin (Shanghai, China). Carbonyl cyanide 3-chlorophenylhydrazone (CCCP, ≥97% (TLC)) was purchased from Solarbio (Beijing, China). All tested strains (*Staphylococcus aureus* CMCC 26003^T^ and *Escherichia coli* CMCC 44102^T^) were purchased from the National Center for Medical Culture Collections (CMCC). Bacterial membrane permeability and membrane potential data were measured and analyzed using an Accuri™ C6 Plus flow cytometer (BD Biosciences, United States), FlowJo™ v10.8 software (BD Life Sciences), and OriginPro (OriginLab Corp. Northampton, MA).

### 3.2 Chemistry

#### 3.2.1 General synthetic procedure for the synthesis of 3a–3j (GPA)

To a stirred solution of methyl ketone (**2**) (1.0 eq) and aldehyde (**1a**–**1j**) (3.0 eq) in ethanol (0.1 M) was added 5 N NaOH (50.0 eq) at 0°C, slowly warmed to RT, and stirred for 24 h. The reaction mixture was diluted with ethyl acetate and brine. Phases were separated, and the aqueous layer was extracted with ethyl acetate. Combined organic layers were dried over Na_2_SO_4_ and concentrated in a vacuum. The residue was purified by column chromatography on the silica gel (ethyl acetate and petroleum ether) to afford the desired product (**3a**–**3j**).

#### 3.2.2 General synthetic procedure for the synthesis of 4a–4j (GPB)

TsOH (2.0–5.0 eq) was added to a solution of **3a**–**3j** (1.0 eq) in THF–MeOH (0.03 M, 1:1, v/v). The reaction mixture was stirred at 50°C for 10 h. The reaction mixture was diluted with ethyl acetate and brine. Phases were separated, and the aqueous layer was extracted with ethyl acetate. Combined organic layers were dried over Na_2_SO_4_ and concentrated in a vacuum. The residue was purified by column chromatography on silica gel (ethyl acetate and petroleum ether) to afford the desired product (**4a**–**4j**).

(E)-3-(2,4-bis(methoxymethoxy)phenyl)-1-(2-hydroxy-3-(2-hydroxy-3-methylbut-3-en-1-yl)-4-(methoxymethoxy)phenyl)prop-2-en-1-one (**3a**): yellow oil, yield: (480 mg, 90%). IR (KBr) *ν*_max_ 2933, 1629, 1234, 1153, 797, and 659 cm^−1^. ^1^H NMR (400 MHz, CDCl_3_) *δ* 13.89 (brs, 1H), 8.19 (d, *J* = 15.5 Hz, 1H), 7.80 (d, *J* = 8.9 Hz, 1H), 7.62 (d, *J* = 3.6 Hz, 1H), 7.59 (s, 1H), 6.86 (s, 1H), 6.79–6.70 (m, 2H), 5.27 (s, 2H), 5.27 (s, 2H), 5.20 (s, 2H), 4.99 (s, 1H), 4.82 (s, 1H), 4.31 (d, *J* = 5.5 Hz, 1H), 3.52 (s, 3H), 3.49 (s, 3H), 3.49 (s, 3H), 3.08 (dd, *J* = 13.6, 3.0 Hz, 1H), 2.98 (dd, *J* = 13.5, 8.8 Hz, 1H), and 1.87 (s, 3H). ^13^C NMR (101 MHz, CDCl_3_) *δ* 193.0, 163.7, 161.2, 160.8, 158.1, 147.8, 140.2, 130.3, 129.6, 118.8, 118.5, 115.8, 115.3, 110.3, 109.5, 105.1, 103.4, 94.8, 94.4, 94.2,75.9, 56.6, 56.6, 56.4, 29.7, and 18.2. HRMS (ESI) calculated for [M–H]^–^ C_26_H_31_O_9_^−^ 487.1974 found 487.1973.

(E)-1-(2,4-dihydroxy-3-(2-hydroxy-3-methylbut-3-en-1-yl)phenyl)-3-(2,4-dihydroxyphenyl)prop-2-en-1-one (**4a**):yellow oil, yield: (200 mg, 70%). IR (KBr) *ν*_max_ 2923, 1607, 1234, 1109, 984, and 790 cm^−1^. ^1^H NMR (400 MHz, CD_3_OD) *δ* 8.12 (d, *J* = 15.4 Hz, 1H), 7.82 (d, *J* = 8.9 Hz, 1H), 7.74 (d, *J* = 15.4 Hz, 1H), 7.54 (d, *J* = 8.3 Hz, 1H), 6.46 (d, *J* = 8.8 Hz, 1H), 6.41–6.38 (m, 2H), 4.82 (s, 1H), 4.74 (s, 1H), 4.43 (t, *J* = 6.4 Hz, 1H), 3.07 (dd, *J* = 13.4, 5.7 Hz, 1H), 2.90 (dd, *J* = 13.5, 7.3 Hz, 1H), and 1.86 (s, 3H). ^13^C NMR (101 MHz, CD_3_OD) *δ* 194.4, 165.5, 164.3, 162.8, 160.8, 148.7, 141.9, 132.4, 130.8, 117.7, 115.6, 114.6, 113.9, 111.0, 109.1, 108.5, 103.5, 76.5, 30.0, and 17.8. HRMS (ESI) calculated for [M–H]^–^ C_20_H_19_O_6_^−^ 355.1187 found 355.1187.

(E)-3-(3,4-bis(methoxymethoxy)phenyl)-1-(2-hydroxy-3-(2-hydroxy-3-methylbut-3-en-1-yl)-4-(methoxymethoxy)phenyl)prop-2-en-1-one (**3b**):yellow oil, yield: (500 mg, 56%). IR (KBr) *ν*_max_ 2925, 1635, 1255, 1154, 996, and 798 cm^−1^. ^1^H NMR (400 MHz, acetone-*d*_6_) *δ* 13.81 (brs, 1H), 8.09 (d, *J* = 9.0 Hz, 1H), 7.86–7.84 (m, 2H), 7.64 (s, 1H), 7.48 (d, *J* = 8.4 Hz, 1H), 7.22 (d, *J* = 8.3 Hz, 1H), 6.76 (d, *J* = 9.0 Hz, 1H), 5.33 (s, 2H), 5.29 (s, 2H), 5.29 (s, 2H), 4.73 (s, 1H), 4.65 (s, 1H), 4.45–4.35 (m, 1H), 3.67 (d, *J* = 4.2 Hz, 1H), 3.50 (s, 3H), 3.49 (s, 3H), 3.48 (s, 3H), 3.01 (dd, *J* = 12.9, 6.4 Hz, 1H), 2.94 (dd, *J* = 13.0, 7.5 Hz, 1H), and 1.83 (s, 3H). ^13^C NMR (101 MHz, acetone-*d*_6_) *δ* 193.5, 164.7, 162.7, 151.2, 149.4, 148.4, 145.1, 130.8, 130.1, 125.3, 120.0, 118.4, 117.6, 116.4, 115.5, 110.1, 106.0, 96.3, 95.8, 95.0, 75.3, 56.6, 56.5, 56.4,30.4, and 17.7. HRMS (ESI) calculated for [M–H]^–^ C_26_H_31_O_9_^−^ 487.1974 found 487.1974.

(E)-1-(2,4-dihydroxy-3-(2-hydroxy-3-methylbut-3-en-1-yl)phenyl)-3-(3,4-dihydroxyphenyl)prop-2-en-1-one (**4b**): yellow oil, yield: (180 mg, 50%). IR (KBr) *ν*_max_ 2925, 1601, 1277, 1107,802, and 541 cm^−1^. ^1^H NMR (400 MHz, Acetone-*d*_6_) *δ* 14.25 (brs, 1H), 9.97 (brs, 1H), 8.66 (brs, 1H), 8.19 (brs, 1H), 8.03 (d, *J* = 8.9 Hz, 1H), 7.78 (d, *J* = 15.3 Hz, 1H), 7.72 (d, *J* = 15.3 Hz, 1H), 7.36 (d, *J* = 1.9 Hz, 1H), 7.24 (dd, *J* = 8.2, 1.9 Hz, 1H), 6.91 (d, *J* = 8.2 Hz, 1H), 6.48 (d, *J* = 8.9 Hz, 1H), 5.36 (d, *J* = 2.8 Hz, 1H), 4.94 (s, 1H), 4.76 (s, 1H), 4.45–4.36 (m, 1H), 3.11 (dd, *J* = 14.3, 3.2 Hz, 1H), 2.91–2.85 (m, 1H), and 1.83 (s, 3H). ^13^C NMR (101 MHz, Acetone-*d*_6_) *δ* 193.0, 165.5, 164.6, 149.2, 148.4, 146.4, 145.5, 131.0, 128.3, 123.5, 118.5, 116.4, 116.0, 114.2, 114.0, 110.3, 109.2, 76.7, 29.5, and 18.4. HRMS (ESI) Calculated for [M–H]^–^ C_20_H_19_O_6_^−^ 355.1187 found 355.1184.

(E)-1-(2-hydroxy-3-(2-hydroxy-3-methylbut-3-en-1-yl)-4-(methoxymethoxy)phenyl)-3-(4-methoxy-3-(methoxymethoxy)phenyl)prop-2-en-1-one (**3c**): yellow oil, yield: (400 mg, 55%). IR (KBr) *ν*_max_ 2959, 1633, 1563, 1267, 1115, 788, and 581 cm^−1^. ^1^H NMR (400 MHz, CDCl_3_) *δ* 13.79 (brs, 1H), 7.84 (d, *J* = 11.8 Hz, 1H), 7.81 (d, *J* = 5.5 Hz, 1H), 7.48 (s, 1H), 7.44 (d, *J* = 15.4 Hz, 1H), 7.30 (d, *J* = 8.4 Hz, 1H), 6.93 (d, *J* = 8.4 Hz, 1H), 6.74 (d, *J* = 9.0 Hz, 1H), 5.29 (s, 2H), 5.28 (s, 2H), 4.99 (s, 1H), 4.82 (s, 1H), 4.31 (dd, *J* = 8.7, 3.7 Hz, 1H), 3.93 (s, 3H), 3.55 (s, 3H), 3.49 (s, 3H), 3.07 (dd, *J* = 13.6, 3.8 Hz, 1H), 2.97 (dd, *J* = 13.6, 8.8 Hz, 1H), and 1.87 (s, 3H). ^13^C NMR (101 MHz, CDCl_3_) *δ* 192.6, 163.7, 161.3, 152.4, 147.9, 147.0, 144.9, 129.7, 128.0, 124.9, 118.3, 115.8, 115.6, 115.2, 111.8, 110.3, 105.2, 95.7, 94.3, 75.9, 56.6, 56.5, 56.2, 29.8, and 18.2. HRMS (ESI) calculated for [M–H]^–^ C_25_H_29_O_8_^−^ 457.1868 found 457.1867.

(E)-1-(2,4-dihydroxy-3-(2-hydroxy-3-methylbut-3-en-1-yl)phenyl)-3-(3-hydroxy-4-methoxyphenyl)prop-2-en-1-one (**4c**): yellow solid, yield: (210 mg, 65%). M.P. 181.7–183.6°C. IR (KBr) *ν*_max_ 2925, 1615, 1510, 1266, 1109, 978, 790, and 514 cm^−1^. ^1^H NMR (400 MHz, Acetone-*d*_6_) *δ* 14.18 (brs, 1H), 8.04 (d, *J* = 8.9 Hz, 1H), 7.78–7.75 (m, 2H), 7.37 (d, *J* = 1.8 Hz, 1H), 7.24 (dd, *J* = 8.3, 1.8 Hz, 1H), 6.99 (d, *J* = 8.3 Hz, 1H), 6.45 (d, *J* = 8.9 Hz, 1H), 4.91 (s, 1H), 4.73 (s, 1H), 4.38 (dd, *J* = 8.0, 3.0 Hz, 1H), 3.88 (s, 3H), 3.08 (dd, *J* = 14.3, 3.3 Hz, 1H), 2.88 (dd, *J* = 14.3, 3.3 Hz, 1H), and 1.80 (s, 3H). ^13^C NMR (101 MHz, Acetone-*d*_6_) *δ* 193.0, 165.5, 164.6, 151.0, 148.4, 147.8, 145.2, 131.2, 129.2, 123.5, 119.3, 114.9, 114.2, 114.0, 112.2, 110.3, 109.3, 76.7, 56.3, 29.7, and 18.4. HRMS (ESI) calculated for [M + H]^+^ C_21_H_23_O_6_^+^ 371.1489 found 371.1488.

(E)-1-(2,4-dihydroxy-3-(2-hydroxy-3-methylbut-3-en-1-yl)phenyl)-3-(4-hydroxy-3-methoxyphenyl)prop-2-en-1-one (**4d**): yellow oil, (250 mg, 38% over two steps). IR (KBr) *ν*_max_ 2925, 1611, 1510, 1266, 1130, 978, 799, and 626 cm^−1^. ^1^H NMR (400 MHz, CDCl_3_) *δ* 13.91 (brs, 1H), 9.18 (brs, 1H), 7.77 (d, *J* = 15.4 Hz, 1H), 7.73 (d, *J* = 9.0 Hz, 1H), 7.44 (d, *J* = 15.4 Hz, 1H), 7.27 (s, 1H), 7.12 (dd, *J* = 8.3, 1.7 Hz, 1H), 6.86 (d, *J* = 8.3 Hz, 1H), 6.52 (d, *J* = 8.9 Hz, 1H), 5.76 (brs, 1H), 5.01 (s, 1H), 4.87 (s, 1H), 4.40 (d, *J* = 8.1 Hz, 1H), 3.93 (s, 3H), 3.21 (dd, *J* = 14.8, 1.6 Hz, 1H), 2.87 (dd, *J* = 14.9, 8.5 Hz, 1H), and 1.86 (s, 3H). ^13^C NMR (101 MHz, CDCl_3_) *δ* 192.2, 164.5, 163.4, 149.0, 147.0, 146.0, 144.2, 130.0, 128.6, 123.0, 118.8, 113.8, 113.3, 113.2, 110.7, 110.6, 109.2, 77.6, 56.2, 28.7, and 18.6. HRMS (ESI) calculated for [M + H]^+^ C_21_H_23_O_6_^+^ 371.1489 found 371.1488.

(E)-1-(2,4-dihydroxy-3-(2-hydroxy-3-methylbut-3-en-1-yl)phenyl)-3-(3,4-dimethoxyphenyl)prop-2-en-1-one (**4e**): Yellow oil, (200 mg, 42% over two steps). IR (KBr) *ν*_max_ 2929, 1630, 1600, 1372, 1262, 1023, and 801 cm^−1^. ^1^H NMR (400 MHz, Acetone-*d*_6_) *δ* 14.20 (brs, 1H), 7.97 (d, *J* = 8.9 Hz, 1H), 7.82–7.80 (m, 2H), 7.50 (s, 1H), 7.34 (d, *J* = 8.3 Hz, 1H), 7.00 (d, *J* = 8.3 Hz, 1H), 6.43 (d, *J* = 8.9 Hz, 1H), 4.91 (s, 1H), 4.73 (s, 1H), 4.38 (dd, *J* = 8.0, 2.9 Hz, 1H), 3.86 (s, 3H), 3.84 (s, 3H), 3.08 (dd, *J* = 14.3, 3.2 Hz, 1H), 2.87 (dd, *J* = 14.3, 3.2 Hz, 1H), and 1.80 (s, 3H). ^13^C NMR (101 MHz, Acetone-*d*_6_) *δ* 193.0, 165.5, 164.6, 153.0, 150.7, 148.3, 145.3, 131.1, 128.8, 124.8, 119.1, 114.2, 114.1, 112.4, 111.7, 110.3, 109.3, 76.7, 56.2, 56.1, 29.7, and 18.4. HRMS (ESI) calculated for [M–H]^–^ C_22_H_23_O_6_^−^ 383.1500, found 383.1501.

(E)-3-(2,3-dihydrobenzo[b][1,4]dioxin-6-yl)-1-(2-hydroxy-3-(2-hydroxy-3-methylbut-3-en-1-yl)-4-(methoxymethoxy)phenyl)prop-2-en-1-one (**3f**): yellow oil, yield: (840 mg, 80%). IR (KBr) *ν*_max_ 2929, 1732, 1566, 1283, 1111, 797, and 557 cm^−1^. ^1^H NMR (400 MHz, CDCl_3_) *δ* 13.79 (brs, 1H), 7.81 (d, *J* = 7.3 Hz, 1H), 7.78 (s, 1H), 7.44 (d, *J* = 15.4 Hz, 1H), 7.20 (s, 1H), 7.17 (d, *J* = 8.7 Hz, 1H), 6.91 (d, *J* = 8.3 Hz, 1H), 6.73 (d, *J* = 9.0 Hz, 1H), 5.36–5.20 (m, 2H), 4.99 (s, 1H), 4.83 (s, 1H), 4.31 (s, 1H), 4.31–4.30 (m, 4H), 3.50 (s, 3H), 3.08 (dd, *J* = 13.6, 3.3 Hz, 1H), 2.98 (dd, *J* = 13.6, 8.9 Hz, 1H), and 1.87 (s, 3H). ^13^C NMR (101 MHz, CDCl_3_) *δ* 192.5, 163.8, 161.3, 147.8, 146.3, 144.7, 143.9, 129.7, 128.5, 123.0, 118.4, 118.0, 117.3, 115.8, 115.2, 110.3, 105.2, 94.3, 76.0, 64.8, 64.4, 56.6, 29.7, and 18.3. HRMS (ESI) Calculated for [M–H]^–^ C_24_H_25_O_7_^−^ 425.1606, Found 425.1607.

(E)-3-(2,3-dihydrobenzo[b][1,4]dioxin-6-yl)-1-(2,4-dihydroxy-3-(2-hydroxy-3-methylbut-3-en-1-yl)phenyl)prop-2-en-1-one (**4f**): Yellow solid, Yield: (640 mg, 85%). M.P. 148.2–150.4°C. IR (KBr) *ν*_max_ 2924, 1634, 1565, 1282, 1252, 1107, and 792 cm^−1^. ^1^H NMR (400 MHz, CDCl_3_) *δ* 13.91 (brs, 1H), 9.13 (brs, 1H), 7.78 (s, 1H), 7.74 (d, *J* = 7.5 Hz, 1H), 7.45 (d, *J* = 15.4 Hz, 1H), 7.18 (s, 1H), 7.15 (s, 1H), 6.90 (d, *J* = 8.1 Hz, 1H), 6.52 (d, *J* = 8.8 Hz, 1H), 5.01 (s, 1H), 4.87 (s, 1H), 4.41 (d, *J* = 8.2 Hz, 1H), 4.33–4.23 (m, 4H), 3.21 (d, *J* = 14.9 Hz, 1H), 2.87 (dd, *J* = 14.9, 8.4 Hz, 1H), and 1.87 (s, 3H). ^13^C NMR (101 MHz, CDCl_3_) *δ* 192.1, 164.6, 163.5, 147.0, 146.2, 144.0, 143.9, 130.0, 128.7, 122.8, 118.8, 117.9, 117.2, 113.8, 113.3, 110.6, 109.2, 77.7, 64.8, 64.4, 28.6, and 18.7. HRMS (ESI) calculated for [M–H]^–^ C_22_H_21_O_6_^−^ 381.1344 found 381.1342.

(E)-1-(2-hydroxy-3-(2-hydroxy-3-methylbut-3-en-1-yl)-4-(methoxymethoxy)phenyl)-3-(pyridin-2-yl)prop-2-en-1-one (**3g**): Yellow oil, Yield: (440 mg, 60%). IR (KBr) *ν*_max_ 2917, 1640, 1612, 1360, 1233, 922, and 541 cm^−1^. ^1^H NMR (400 MHz, Acetone-*d*_6_) *δ* 13.60 (brs, 1H), 8.71 (d, *J* = 4.5 Hz, 1H), 8.30 (d, *J* = 15.1 Hz, 1H), 8.07 (d, *J* = 9.1 Hz, 1H), 7.93–7.89 (m, 1H), 7.87 (d, *J* = 11.1 Hz, 1H), 7.81 (d, *J* = 7.7 Hz, 1H), 7.46–7.39 (m, 1H), 6.83 (d, *J* = 9.1 Hz, 1H), 5.36 (s, 2H), 4.73 (s, 1H), 4.65 (s, 1H), 4.42 (d, *J* = 5.2 Hz, 1H), 3.49 (s, 3H), 3.00 (d, *J* = 6.4 Hz, 1H), 2.99–2.91 (m, 1H), and 1.84 (s, 3H). ^13^C NMR (101 MHz, Acetone-*d*_6_) *δ* 193.7, 164.8, 163.1, 154.0, 151.1, 149.4, 144.0, 137.9, 131.1, 126.1, 125.6, 125.0, 116.5, 115.6, 110.2, 106.3, 95.1, 75.2, 56.6, 30.3, and 17.7. HRMS (ESI) calculated for [M–H]^–^ C_21_H_22_NO_5_^−^ 368.1503 found 368.1505.

(E)-1-(2,4-dihydroxy-3-(2-hydroxy-3-methylbut-3-en-1-yl)phenyl)-3-(pyridin-2-yl)prop-2-en-1-one (**4g**): yellow solid, yield: (200 mg, 65%). M.P. 139.5–141°C. IR (KBr) *ν*_max_ 2923, 1644, 1585, 1439, 1236, 1108, and 784 cm^−1^. ^1^H NMR (400 MHz, Acetone-*d*_6_) *δ* 13.96 (brs, 1H), 10.11 (brs, 1H), 8.70 (d, *J* = 4.5 Hz, 1H), 8.28 (d, *J* = 15.1 Hz, 1H), 8.02–7.79 (m, 4H), 7.46–7.38 (m, 1H), 6.54 (d, *J* = 8.9 Hz, 1H), 5.39 (brs, 1H), 4.94 (s, 1H), 4.77 (s, 1H), 4.43 (dd, *J* = 7.8, 2.8 Hz, 1H), 3.11 (dd, *J* = 14.3, 3.3 Hz, 1H), 3.02–2.90 (m, 1H), and 1.84 (s, 3H). ^13^C NMR (101 MHz, Acetone-*d*_6_) *δ* 193.0, 165.6, 165.2, 154.1, 151.1, 148.3, 143.5, 137.9, 131.4, 125.9, 125.5, 125.1, 114.2, 114.1, 110.4, 109.7, 76.6, 29.7, and 18.3. HRMS (ESI) calculated for [M–H]^–^ C_19_H_18_NO_4_^−^ 324.1241 found 324.1243.

(E)-1-(2-hydroxy-3-(2-hydroxy-3-methylbut-3-en-1-yl)-4-(methoxymethoxy)phenyl)-3-(pyridin-3-yl)prop-2-en-1-one (**3h**): yellow oil, yield: (400 mg, 55%). IR (KBr) *ν*_max_ 2924, 1638, 1581, 1240, 1116, 1061, 794, and 707 cm^−1^. ^1^H NMR (400 MHz, CDCl_3_) *δ* 13.51 (brs, 1H), 8.88 (s, 1H), 8.65 (d, *J* = 3.6 Hz, 1H), 7.96 (d, *J* = 7.8 Hz, 1H), 7.87 (d, *J* = 15.5 Hz, 1H), 7.80 (d, *J* = 9.0 Hz, 1H), 7.66 (d, *J* = 15.5 Hz, 1H), 7.44–7.35 (m, 1H), 6.76 (d, *J* = 9.0 Hz, 1H), 5.33–5.25 (m, 2H), 4.99 (s, 1H), 4.83 (s, 1H), 4.33 (dd, *J* = 8.7, 3.7 Hz, 1H), 3.50 (s, 3H), 3.08 (dd, *J* = 13.6, 3.8 Hz, 1H), 2.98 (dd, *J* = 13.6, 8.8 Hz, 1H), and 1.86 (s, 3H). ^13^C NMR (101 MHz, CDCl_3_) *δ* 191.9, 163.9, 161.8, 151.3, 150.1, 147.8, 141.0, 135.0, 129.8, 124.0, 122.4, 122.4, 116.0, 114.9, 110.4, 105.5, 94.3, 75.8, 56.7, 29.7, and 18.2. HRMS (ESI) Calculated for [M–H]^–^ C_21_H_22_NO_5_^−^ 368.1503 found 368.1503.

(E)-1-(2,4-dihydroxy-3-(2-hydroxy-3-methylbut-3-en-1-yl)phenyl)-3-(pyridin-3-yl)prop-2-en-1-one (**4h**): yellow oil, yield: (160 mg, 50%). IR (KBr) *ν*_max_ 2923, 1640, 1611, 1357, 1246, 1107, 796, and 640 cm^−1^. ^1^H NMR (400 MHz, DMSO-*d*_6_) *δ* 13.83 (brs, 1H), 10.69 (brs, 1H), 9.03 (s, 1H), 8.61 (d, *J* = 4.6 Hz, 1H), 8.37 (d, *J* = 7.9 Hz, 1H), 8.14–8.09 (m, 2H), 7.82 (d, *J* = 15.5 Hz, 1H), 7.50 (dd, *J* = 7.8, 4.8 Hz, 1H), 6.48 (d, *J* = 8.9 Hz, 1H), 4.99 (brs, 1H), 4.62 (s, 1H), 4.60 (s, 1H), 4.29 (t, *J* = 6.7 Hz, 1H), 2.83 (dd, *J* = 13.0, 6.8 Hz, 1H), 2.74 (dd, *J* = 13.0, 7.0 Hz, 1H), and 1.73 (s, 3H). ^13^C NMR (101 MHz, DMSO-*d*_6_) *δ* 191.4, 164.5, 163.8, 151.0, 150.5, 148.1, 140.1, 135.3, 130.5, 124.0, 123.9, 123.2, 112.6, 112.5, 109.9, 107.7, 73.3, 29.0, and 17.3. HRMS (ESI) calculated for [M–H]^–^ C_19_H_18_NO_4_^−^ 324.1241 found 324.1242.

(E)-1-(2-hydroxy-3-(2-hydroxy-3-methylbut-3-en-1-yl)-4-(methoxymethoxy)phenyl)-3-(thiophen-2-yl)prop-2-en-1-one (**3i**): yellow solid, yield: (460 mg, 70%). M.P. 82.1–83.8 °C. IR (KBr) *ν*_max_ 2914, 1627, 1563, 1113, 1055, 793, and 714 cm^−1^. ^1^H NMR (400 MHz, CDCl_3_) *δ* 13.71 (brs, 1H), 8.01 (d, *J* = 15.1 Hz, 1H), 7.76 (d, *J* = 9.0 Hz, 1H), 7.45 (d, *J* = 5.0 Hz, 1H), 7.38–7.34 (m, 2H), 7.10 (t, *J* = 4.3 Hz, 1H), 6.73 (d, *J* = 9.0 Hz, 1H), 5.39–5.18 (m, 2H), 4.99 (s, 1H), 4.82 (s, 1H), 4.31 (dd, *J* = 8.7, 3.7 Hz, 1H), 3.49 (s, 3H), 3.07 (dd, *J* = 13.6, 3.8 Hz, 1H), 2.97 (dd, *J* = 13.6, 8.8 Hz, 1H), and 1.86 (s, 3H). ^13^C NMR (101 MHz, CDCl_3_) *δ* 192.0, 163.7, 161.5, 147.8, 140.4, 137.3, 132.6, 129.6, 129.4, 128.6, 119.1, 115.8, 115.0, 110.3, 105.3, 94.3, 75.9, 56.6, 29.7, and 18.2. HRMS (ESI) calculated for [M–H]^–^ C_20_H_21_SO_5_^−^ 373.1115 found 373.1117.

(E)-1-(2,4-dihydroxy-3-(2-hydroxy-3-methylbut-3-en-1-yl)phenyl)-3-(thiophen-2-yl)prop-2-en-1-one (**4i**): Yellow oil, Yield: (260 mg, 80%). IR (KBr) *ν*_max_ 2920, 1623, 1565, 1374, 1277, 1106, 799, and 706 cm^−1^. ^1^H NMR (400 MHz, CDCl_3_) *δ* 13.85 (brs, 1H), 9.22 (brs, 1H), 7.98 (d, *J* = 15.1 Hz, 1H), 7.71 (d, *J* = 8.9 Hz, 1H), 7.43 (d, *J* = 5.0 Hz, 1H), 7.37 (d, *J* = 10.9 Hz, 1H), 7.35 (s, 1H), 7.09 (t, *J* = 4.3 Hz, 1H), 6.52 (d, *J* = 8.9 Hz, 1H), 5.00 (s, 1H), 4.87 (s, 1H), 4.40 (d, *J* = 8.4 Hz, 1H), 2.89 (d, *J* = 8.3 Hz, 1H), 2.85 (d, *J* = 8.2 Hz, 1H), and 1.86 (s, 3H). ^13^C NMR (101 MHz, CDCl_3_) *δ* 191.6, 164.5, 163.6, 146.9, 140.5, 136.7, 132.3, 130.0, 129.1, 128.5, 119.5, 113.6, 113.3, 110.6, 109.3, 77.6, 28.6, and 18.6. HRMS (ESI) calculated for [M–H]^–^ C_18_H_17_SO_4_^−^ 329.0853 found 329.0852.

(E)-1-(2-hydroxy-3-(2-hydroxy-3-methylbut-3-en-1-yl)-4-(methoxymethoxy)phenyl)-3-(thiophen-3-yl)prop-2-en-1-one (**3j**): Yellow oil, Yield: (560 mg, 75%). IR (KBr) *ν*_max_ 2925, 1631, 1568, 1269, 1111, 939, and 784 cm^−1^. ^1^H NMR (400 MHz, CDCl_3_) *δ* 13.71 (brs, 1H), 7.89 (d, *J* = 15.3 Hz, 1H), 7.78 (d, *J* = 9.0 Hz, 1H), 7.64 (s, 1H), 7.48–7.35 (m, 3H), 6.73 (d, *J* = 9.0 Hz, 1H), 5.34–5.23 (m, 2H), 4.99 (s, 1H), 4.83 (s, 1H), 4.31 (d, *J* = 7.3 Hz, 1H), 3.49 (s, 3H), 3.08 (dd, *J* = 13.6, 3.7 Hz, 1H), 2.98 (dd, *J* = 13.7, 8.8 Hz, 1H), and 1.86 (s, 3H). ^13^C NMR (101 MHz, CDCl_3_) *δ* 192.8, 163.7, 161.5, 147.8, 138.3, 138.2, 129.8, 129.7, 127.3, 125.4, 120.0, 115.9, 115.1, 110.3, 105.2, 94.3, 75.9, 56.6, 29.7, and 18.2. HRMS (ESI) calculated for [M–H]^–^ C_20_H_21_SO_5_^−^ 373.1115 found 373.1116.

(E)-1-(2,4-dihydroxy-3-(2-hydroxy-3-methylbut-3-en-1-yl)phenyl)-3-(thiophen-3-yl)prop-2-en-1-one (**4j**): yellow oil, yield: (260 mg, 65%). IR (KBr) *ν*_max_ 2915, 1629, 1569, 1277, 1106, 785, and 584 cm^−1^. ^1^H NMR (400 MHz, CDCl_3_) *δ* 13.85 (brs, 1H), 9.24 (brs, 1H), 7.85 (d, *J* = 15.3 Hz, 1H), 7.71 (d, *J* = 8.9 Hz, 1H), 7.60 (d, *J* = 1.8 Hz, 1H), 7.42–7.36 (m, 3H), 6.51 (d, *J* = 8.9 Hz, 1H), 5.01 (s, 1H), 4.87 (s, 1H), 4.40 (d, *J* = 8.2 Hz, 1H), 2.95 (d, *J* = 2.6 Hz, 1H), 2.87 (dd, *J* = 14.8, 8.5 Hz, 1H), and 1.86 (s, 3H). ^13^C NMR (101 MHz, CDCl_3_) *δ* 192.3, 164.5, 163.5, 146.9, 138.3, 137.7, 130.0, 129.4, 127.2, 125.3, 120.3, 113.7, 113.3, 110.6, 109.2, 77.6, 28.6, and 18.6. HRMS (ESI) calculated for [M–H]^–^ C_18_H_17_SO_4_^−^ 329.0853 found 329.0854.

### 3.3 *In vitro* antibacterial activity

#### 3.3.1 Minimum inhibitory concentration test

The MIC of sanjuanolide, curcumin, and the synthesized chalcone derivatives (**4a**–**4j**) were evaluated against one Gram-positive bacterial strain *S. aureus* CMCC 26003 and one Gram-negative bacterial strain *E*. *coli* CMCC 44102 in Mueller–Hinton broth (MHB) medium (Li et al., 2019). Since sanjuanolide showed the best bacteriostatic effect (MIC = 12.5 μg/ml, the same as **4c**), the subsequent *in vitro* mesoscopic effects of experiments of antibacterial activities were mainly focused on natural products like sanjuanolide.

#### 3.3.2 Antibacterial effect of sanjuanolide determined on the MHA plate

To evaluate the effect of sanjuanolide on bacteria, single colonies of *E*. *coli* CMCC 44102 and *S. aureus* CMCC 26003 were incubated in 5 ml of MHB medium and shaken at 200 rpm and at 37 °C for 12 h. A measure of 100 μl of the fresh bacterial solution was spread on MHA medium containing 12.5 μg/ml of sanjuanolide. MHA medium without the addition of sanjuanolide was used as a control. Samples were taken and gradiently diluted after incubation for 6 and 12 h. The growth status of bacteria on the plates was observed.

#### 3.3.3 SEM analyses of bacterial cell surface morphology

In order to visually verify the antibacterial effect of sanjuanolide, the micromorphological changes of *E*. *coli* CMCC 44102 and *S. aureus* CMCC 26003 affected by sanjuanolide were observed by SEM (Quanta FEG250, FEI). Cells were cultured in LB medium supplemented with 12.5 μg/ml sanjuanolide for 12 h. Raw cells and sanjuanolide-exposed cells were separately fixed in 2.5% glutaraldehyde and then dehydrated in a graded ethanol series (30–100%). Lyophilized raw control and sanjuanolide-exposed cells were mounted on an aluminum stub using carbon tape and coated with gold under vacuum in an argon atmosphere. The surface microstructure of the samples was visualized by SEM at a voltage of 20 kV.

#### 3.3.4 Cytometric analysis of the effect of sanjuanolide on membrane permeability

Cells of *E*. *coli* CMCC 44102 and *S. aureus* CMCC 26003 were inoculated into 5 ml LB medium to make the initial inoculum amount about 1 × 10^6^ CFU/ml. After culturing at 37 °C and 180 rpm for 2 h, sanjuanolide was added to make the concentration of 12.5 and 25 μg/ml. Cell suspension without the addition of sanjuanolide was used as a control. All samples were subsequently incubated at 37°C for another 1.5 h. Then, 1 ml of bacterial suspension from each sample was transferred to a 1.5-ml centrifuge tube. Cells were centrifuged twice in phosphate-buffered saline (PBS, pH = 7.4), centrifuged at 10000 r/min for 10 min, and then resuspended in PBS containing 1 mmol/L ethylenediaminetetraacetic acid (EDTA) to increase the cell membrane’s penetration of fluorescent probes. A measure of 10 μl of the 10 mmol/L cFDA solution was added to each tube and incubated at 40°C for 10 min. After incubation, samples were washed twice with PBS, then suspended in PBS, and kept in an ice bath away from light for following cytometric analysis.

#### 3.3.5 Cytometric analysis of the effect of sanjuanolide on membrane potential

Cells of *E*. *coli* CMCC 44102 and *S. aureus* CMCC 26003 were pretreated in the same way as the membrane permeability experiments. The difference is that 0.5 mmol/L CCCP solution instead of sanjuanolide needs to be added to the sample tube of the positive control group (CCCP + group), in order to minimize the membrane potential. CCCP tubes untreated with sanjuanolide and CCCP were served as negative controls. Then, 10 μl of 3 mmol/L DiOC_2_(3) solution was added to each tube and incubated at room temperature for 25 min. After incubation, samples were washed twice with PBS and then analyzed by flow cytometry.

*E*. *coli* CMCC 44102 and *S. aureus* CMCC 26003 untreated with sanjuanolide or fluorescent dyes were used to localize forward and lateralized bacterial populations, recording 10,000 cells for flow cytometric analysis. MFI was defined as the ratio between bacterial populations and fluorescent standard beads, using the absolute value of the fluorescence intensity. Carboxyfluorescein (CF) was excited at 488 nm and detected at 520 nm (Nguefack et al., 2004). DiOC_2_(3) was excited at 488 nm, and its red and green fluorescence were detected by bandpass filters at 610-nm, 19-nm bandwidth, and 530-nm, 20-nm bandwidth, respectively (Novo et al., 2000). Result analysis and plotting were performed by BD Accuri™ C6 Plus, FlowJo, and Origin software.

## 4 Conclusion

In the broader application, abuse and misuse of antibiotics is becoming an increasingly serious problem that brings about dangerous drug resistance; thus, the development of novel and structurally diverse compounds with potential antimicrobial properties is urgently needed. In this study, sanjuanolide and its 10 new derivatives were efficiently synthesized through combinatorial chemistry, and two of these derivatives (**4c** and **4d**) have obvious antibacterial ability against the *S. aureus* CMCC 26003 strain. Furthermore, natural chalcone sanjuanolide exhibited selective inhibitory activity against clinical Gram-positive *S. aureus* CMCC 26003 strain but did not show significant inhibitory activity against Gram-negative *E*. *coli* CMCC 44102. The results of the *in vitro* antibacterial experiment showed that sanjuanolide changed the cell structure and altered the permeability of the cell membrane and reduced the membrane potential of *S. aureus* CMCC 26003. Therefore, sanjuanolide and its new derivatives might have the potential for further development, especially as an adjuvant for a combination strategy between antibiotics and sanjuanolide.

## Data Availability

The original contributions presented in the study are included in the article/Supplementary Material; further inquiries can be directed to the corresponding authors.

## References

[B1] Ammaji S. Masthanamma S. Bhandare R. R. Annadurai S. Shaik A. B. (2022). Antitubercular and antioxidant activities of hydroxy and chloro substituted chalcone analogues: Synthesis, biological and computational studies. Arab. J. Chem. 15, 103581. 10.1016/j.arabjc.2021.103581

[B2] Chen Z. Zheng C. Sun L. Piao H. (2010). Synthesis of new chalcone derivatives containing a rhodanine-3-acetic acid moiety with potential anti-bacterial activity. Eur. J. Med. Chem. 45 (12), 5739–5743. 10.1016/j.ejmech.2010.09.031 20889240

[B3] Duvauchelle V. Majdi C. Bénimélis D. Dunyach-Remy C. Meffre P. Benfodda Z. (2021). Synthesis, structure elucidation, antibacterial activities, and synergistic effects of novel juglone and naphthazarin derivatives against clinical methicillin-resistant *Staphylococcus aureus* strains. Front. Chem. 9, 773981. 10.3389/fchem.2021.773981 34869221 PMC8640087

[B4] Effenberger F. Jäger J. (1997). Synthesis of the adrenergic bronchodilators (*R*)-Terbutaline and (*R*)-Salbutamol from (*R*)-Cyanohydrins^1^ . J. Org. Chem. 62 (12), 3867–3873. 10.1021/jo970032d

[B5] Hoefel D. Grooby W. L. Monis P. T. Andrews S. Saint C. P. (2003). A comparative study of carboxyfluorescein diacetate and carboxyfluorescein diacetate succinimidyl ester as indicators of bacterial activity. J. Microbiol. Methods 52 (3), 379–388. 10.1016/S0167-7012(02)00207-5 12531507

[B6] Hotsumi M. Tajiri M. Nikaido Y. Sato T. Makabe K. Konno H. (2019). Design, synthesis, and evaluation of a water soluble C5-monoketone type Curcumin analogue as a potent amyloid β aggregation inhibitor. Bioorg. Med. Chem. Lett. 29 (16), 2157–2161. 10.1016/j.bmcl.2019.06.052 31262559

[B7] Jogireddy R. Maier M. E. (2006). Synthesis of luminacin D. J. Org. Chem. 71 (18), 6999–7006. 10.1021/jo061104g 16930055

[B8] Kamal A. Reddy J. S. Ramaiah M. J. Dastagiri E. Bharathi E. V. Sagar M. V. P. (2010). Design, synthesis and biological evaluation of imidazopyridine/pyrimidine-chalcone derivatives as potential anticancer agents. Medchemcomm 1 (5), 355. 10.1039/c0md00116c

[B9] Kasetti A. B. Singhvi I. Nagasuri R. Bhandare R. R. Shaik A. B. (2021). Thiazole–chalcone hybrids as prospective antitubercular and antiproliferative agents: Design, synthesis, biological, molecular docking studies and in silico ADME evaluation. Molecules 26, 2847. 10.3390/molecules26102847 34064806 PMC8151732

[B10] Kim M. S. Ki Y. Ahn S. Y. Yoon S. Kim S. E. Park H. G. (2014). Asymmetric synthesis and receptor activity of chiral simplified resiniferatoxin (sRTX) analogues as transient receptor potential vanilloid 1 (TRPV1) ligands. Bioorg. Med. Chem. Lett. 24 (1), 382–385. 10.1016/j.bmcl.2013.10.064 24321344 PMC6957263

[B11] Lagu S. B. Yejella R. P. Bhandare R. R. Shaik A. B. (2020). Design, synthesis, and antibacterial and antifungal activities of novel trifluoromethyl and trifluoromethoxy substituted chalcone derivatives. Pharmaceuticals 13, 375. 10.3390/ph13110375 PMC7695348 33182305

[B12] Li Y. Sun B. Zhai J. Fu L. Zhang S. Zhang J. (2019). Synthesis and antibacterial activity of four natural chalcones and their derivatives. Tetrahedron Lett. 60 (43), 151165. 10.1016/j.tetlet.2019.151165

[B13] Liegault B. Lapointe D. Caron L. Vlassova A. Fagnou K. (2009). Establishment of broadly applicable reaction conditions for the palladium-catalyzed direct arylation of heteroatom-containing aromatic compounds. J. Org. Chem. 74 (5), 1826–1834. 10.1021/jo8026565 19206211

[B14] Morono Y. Takano S. Miyanaga K. Tanji Y. Unno H. Hori K. (2004). Application of glutaraldehyde for the staining of esterase-active cells with carboxyfluorescein diacetate. Biotechnol. Lett. 26 (5), 379–383. 10.1023/B:BILE.0000018255.89810.1a 15104134

[B15] Nguefack J. Budde B. B. Jakobsen M. (2004). Five essential oils from aromatic plants of Cameroon: Their antibacterial activity and ability to permeabilize the cytoplasmic membrane of Listeria innocua examined by flow cytometry. Lett. Appl. Microbiol. 39 (5), 395–400. 10.1111/j.1472-765X.2004.01587.x 15482428

[B16] Nielsen S. F. Larsen M. Boesen T. Schønning K. Kromann H. (2005). Cationic chalcone antibiotics. Design, synthesis, and mechanism of action. J. Med. Chem. 48, 2667–2677. 10.1021/jm049424k 15801857

[B17] Novo D. J. Perlmutter N. G. Hunt R. H. Shapiro H. M. (2000). Multiparameter flow cytometric analysis of antibiotic effects on membrane potential, membrane permeability, and bacterial counts of Staphylococcus aureus and micrococcus luteus. Antimicrob. Agents Chemother. 44 (4), 827–834. 10.1128/AAC.44.4.827-834.2000 10722477 PMC89778

[B18] Novo D. Perlmutter N. G. Hunt R. H. Shapiro H. M. (1999). Accurate flow cytometric membrane potential measurement in bacteria using diethyloxacarbocyanine and a ratiometric technique. Cytometry 35 (1), 55–63. 10.1002/(sici)1097-0320(19990101)35:1<55:aid-cyto8>3.0.co;2-2 10554181

[B19] Ohkatsu Y. Satoh T. (2008). Antioxidant and photo-antioxidant activities of chalcone derivatives. J. Jpn. Pet. Inst. 51 (5), 298–308. 10.1627/jpi.51.298

[B20] Salehi B. Quispe C. Chamkhi I. Omari N. E. Balahbib A. Sharifi-Rad J. (2021). Pharmacological properties of chalcones: A review of preclinical including molecular mechanisms and clinical evidence. Front. Pharmacol. 11, 592654. 10.3389/fphar.2020.592654 33536909 PMC7849684

[B21] Shaffer C. V. Cai S. Peng J. Robles A. J. Hartley R. M. Powell D. R. (2016). Texas native plants yield compounds with cytotoxic activities against prostate cancer cells. J. Nat. Prod. 79 (3), 531–540. 10.1021/acs.jnatprod.5b00908 26785306 PMC4860899

[B22] Vaara M. (1992). Agents that increase the permeability of the outer membrane. Microbiol. Rev. 56, 395–411. 10.1128/mr.56.3.395-411.1992 1406489 PMC372877

[B23] Yang H.-M. Shin H.-R. Cho S.-H. Bang S.-C. Song G.-Y. Ju J.-H. (2007). Structural requirement of chalcones for the inhibitory activity of interleukin-5. Bioorg. Med. Chem. 15, 104–111. 10.1016/j.bmc.2006.10.007 17064909

[B24] Zhai J. Fu L. Li Y. Zhao R. Wang R. Deng H. (2019). Synthesis and biological activities evaluation of sanjuanolide and its analogues. Bioorg. Med. Chem. Lett. 29 (2), 326–328. 10.1016/j.bmcl.2018.11.020 30472027

[B25] Zheng C. Jiang S. Chen Z. Ye B. Piao H. (2011). Synthesis and anti-bacterial activity of some heterocyclic chalcone derivatives bearing thiofuran, furan, and quinoline moieties. Arch. Pharm. Weinh. 344 (10), 689–695. 10.1002/ardp.201100005 21887800

[B26] Zhuang C. Zhang W. Sheng C. Zhang W. Xing C. Miao Z. (2017). Chalcone: A privileged structure in medicinal chemistry. Chem. Rev. 117 (12), 7762–7810. 10.1021/acs.chemrev.7b00020 28488435 PMC6131713

