# Supplementary material for: Structural modification and antibacterial property studies of natural chalcone sanjuanolide
Source: Front Chem. 2022 Aug 5;10:959250. doi: 10.3389/fchem.2022.959250 (PMC9388722; doi:10.3389/fchem.2022.959250)
Supplement: Supplementary file 1 [file DataSheet1.docx]

Supplementary Material

# NMR Spectra


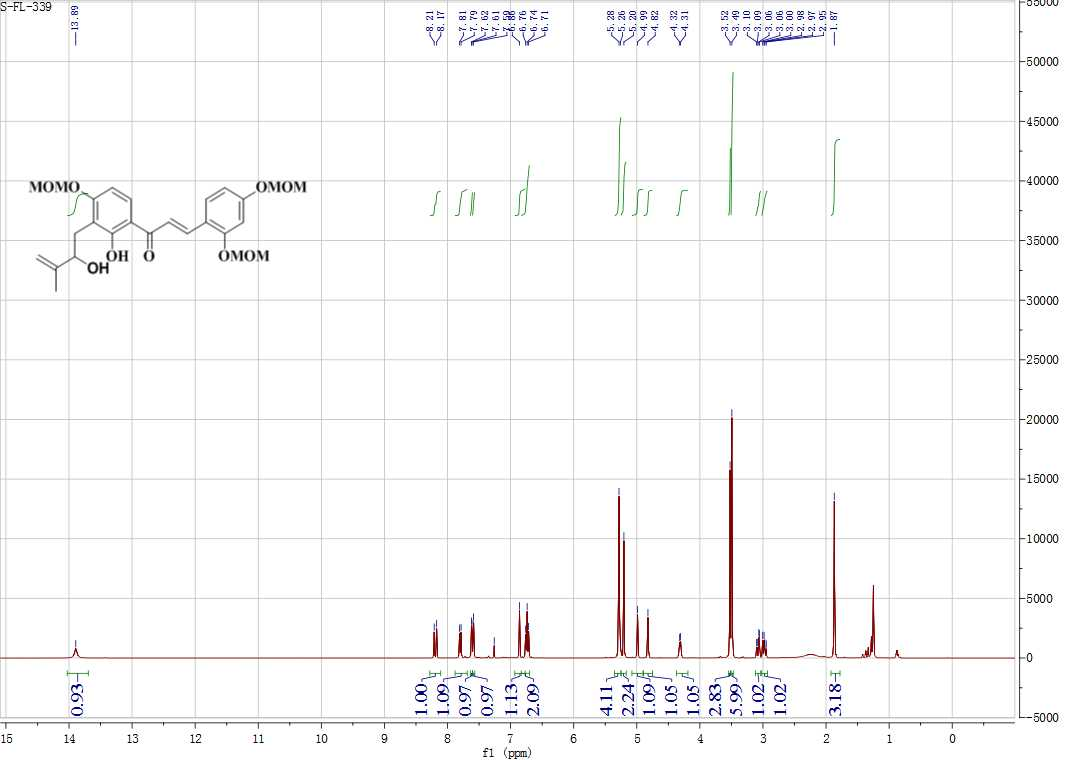


^1^H NMR of **3a**


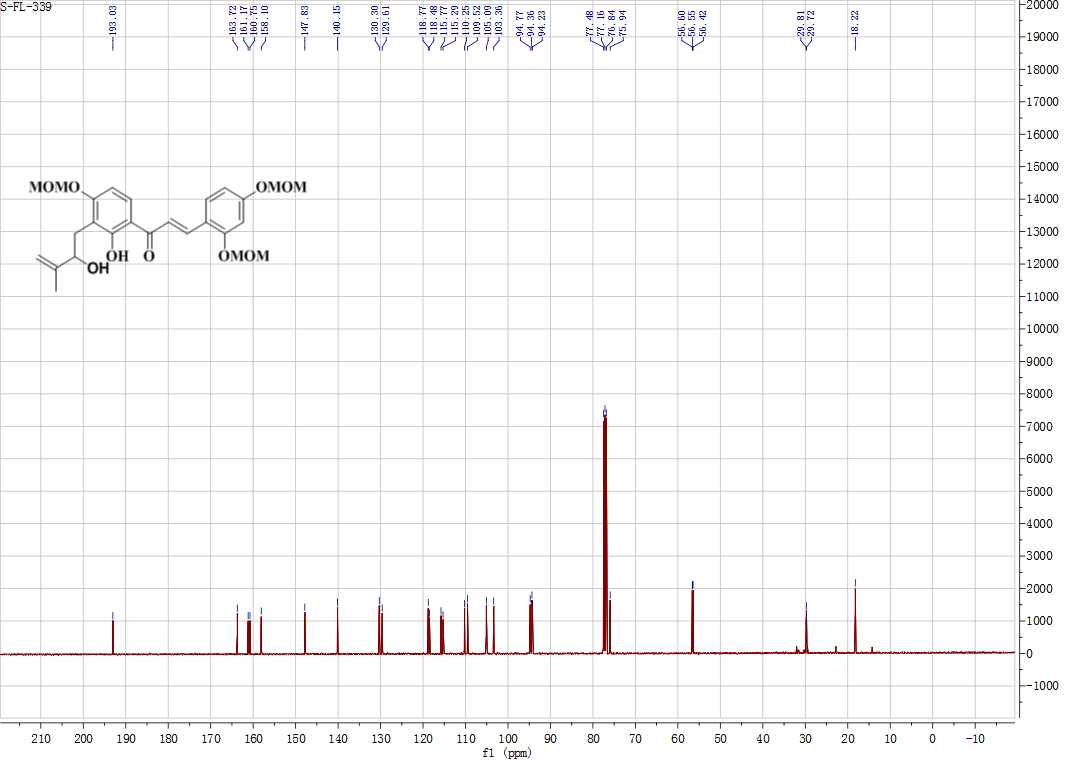


^13^C NMR of **3a**


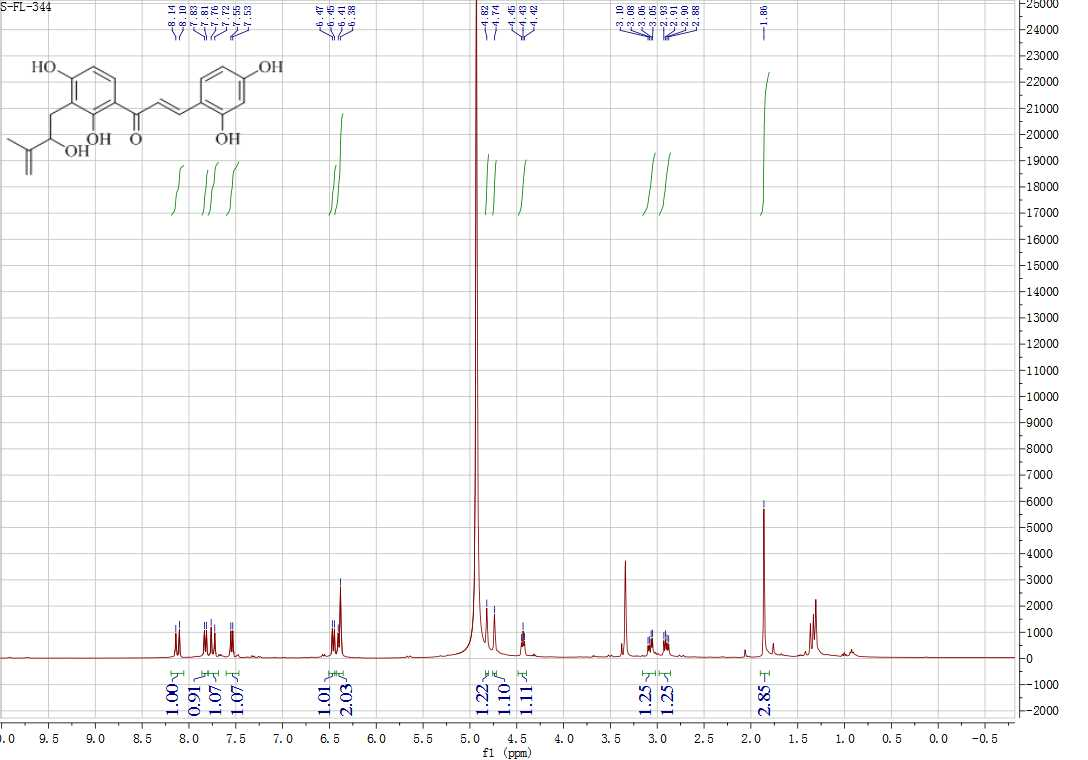


^1^H NMR of **4a**


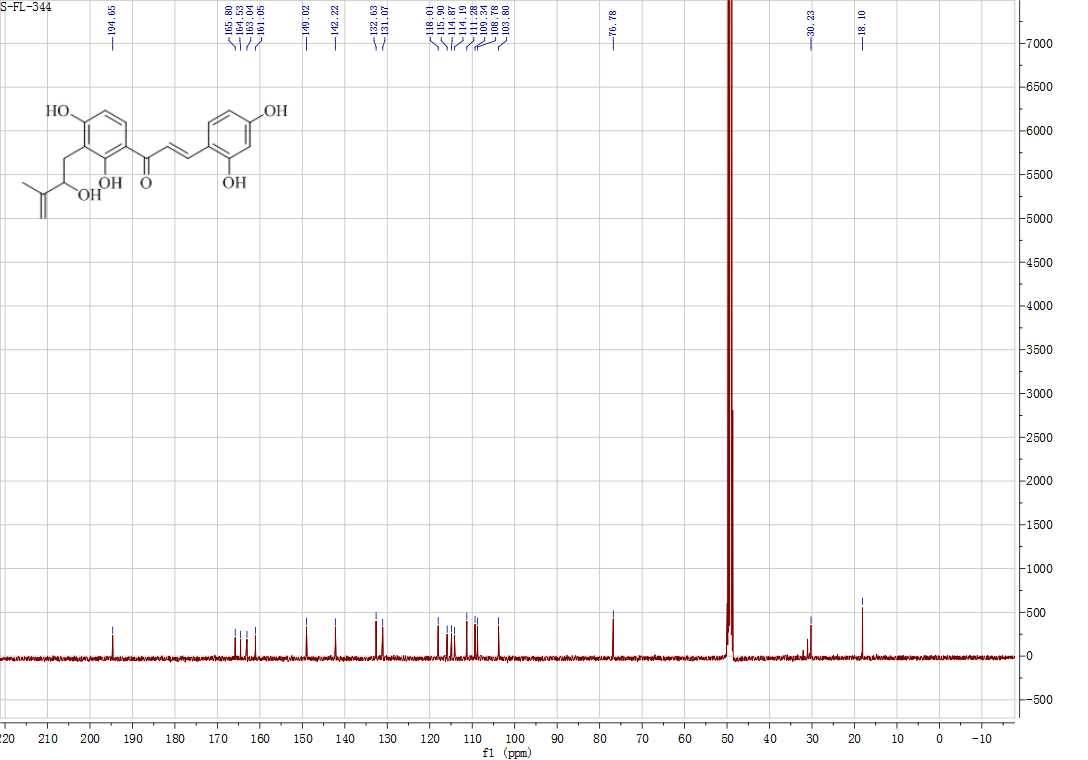


^13^C NMR of **4a**


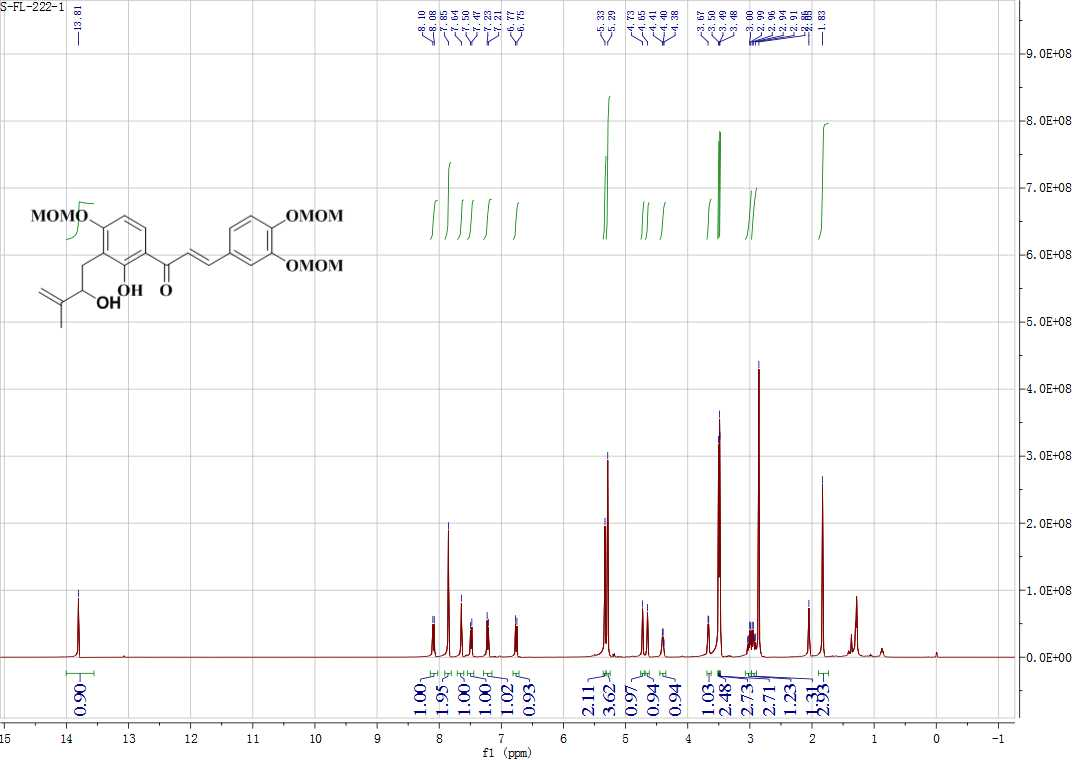


^1^H NMR of **3b**


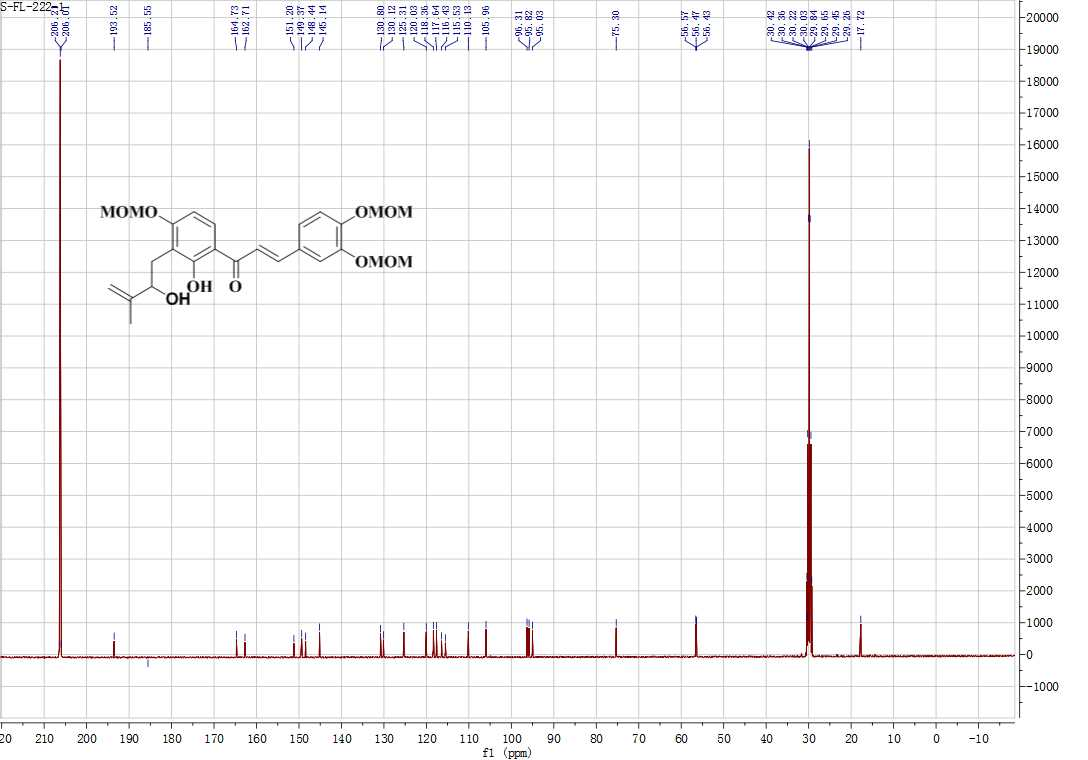


^13^C NMR of **3b**


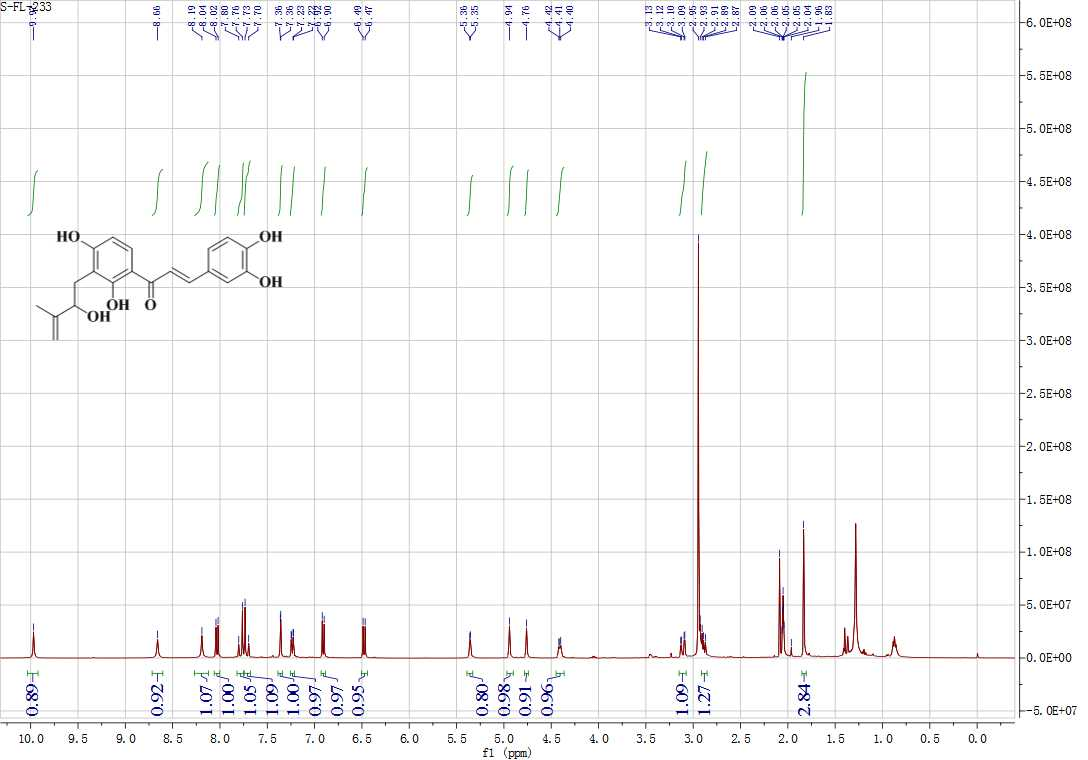


^1^H NMR of **4b**


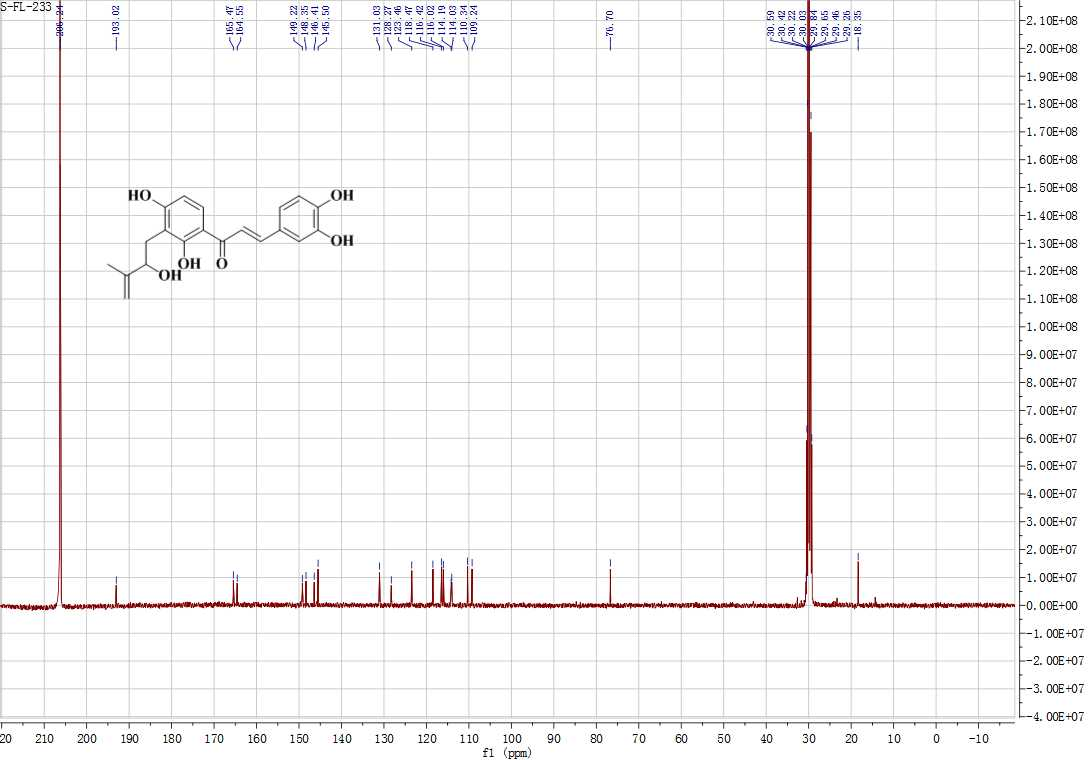


^13^C NMR of **4b**


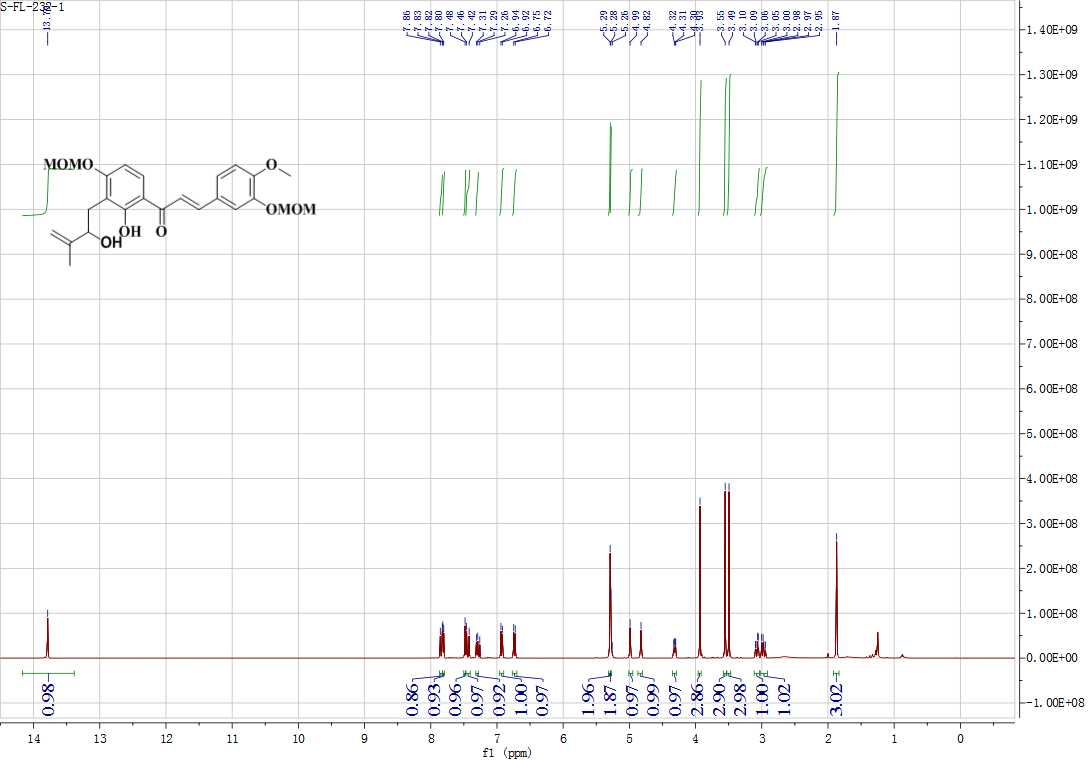


^1^H NMR of **3c**


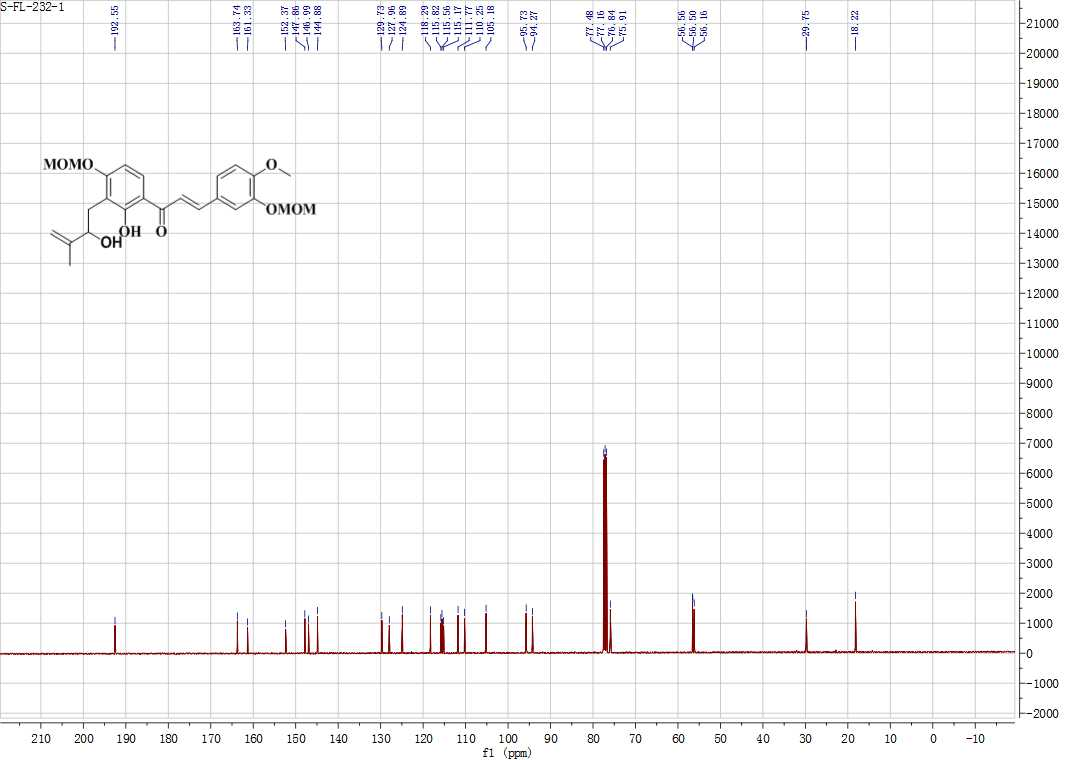


^13^C NMR of **3c**


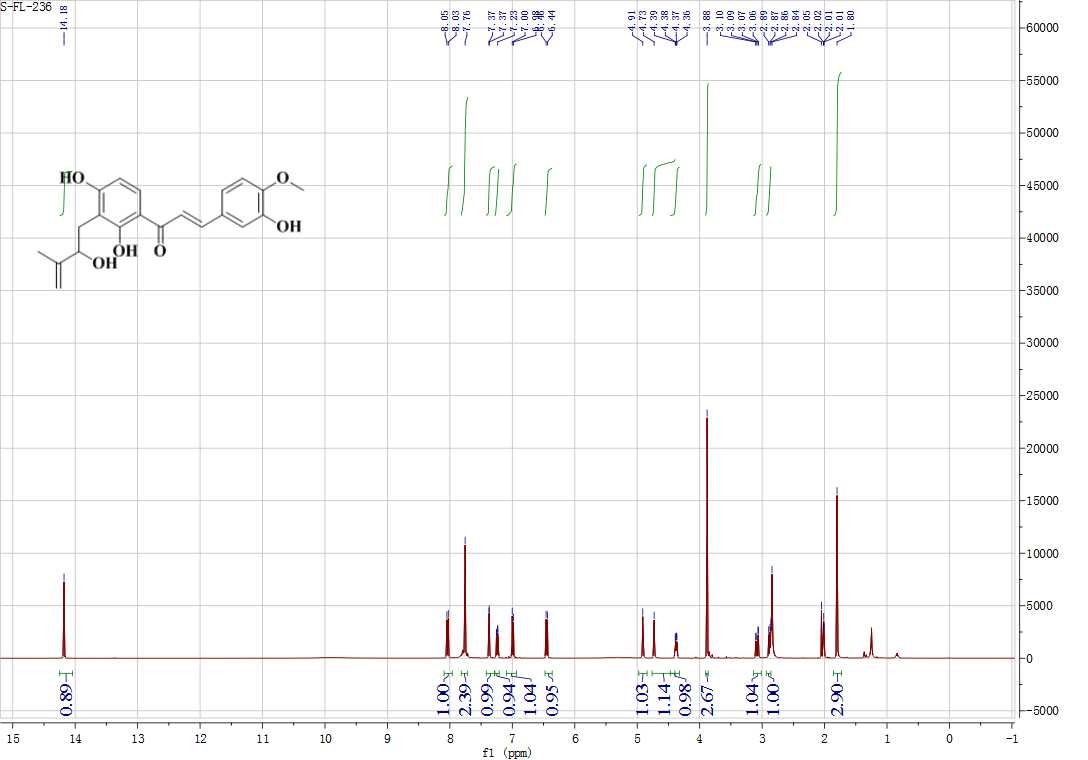


^1^H NMR of **4c**


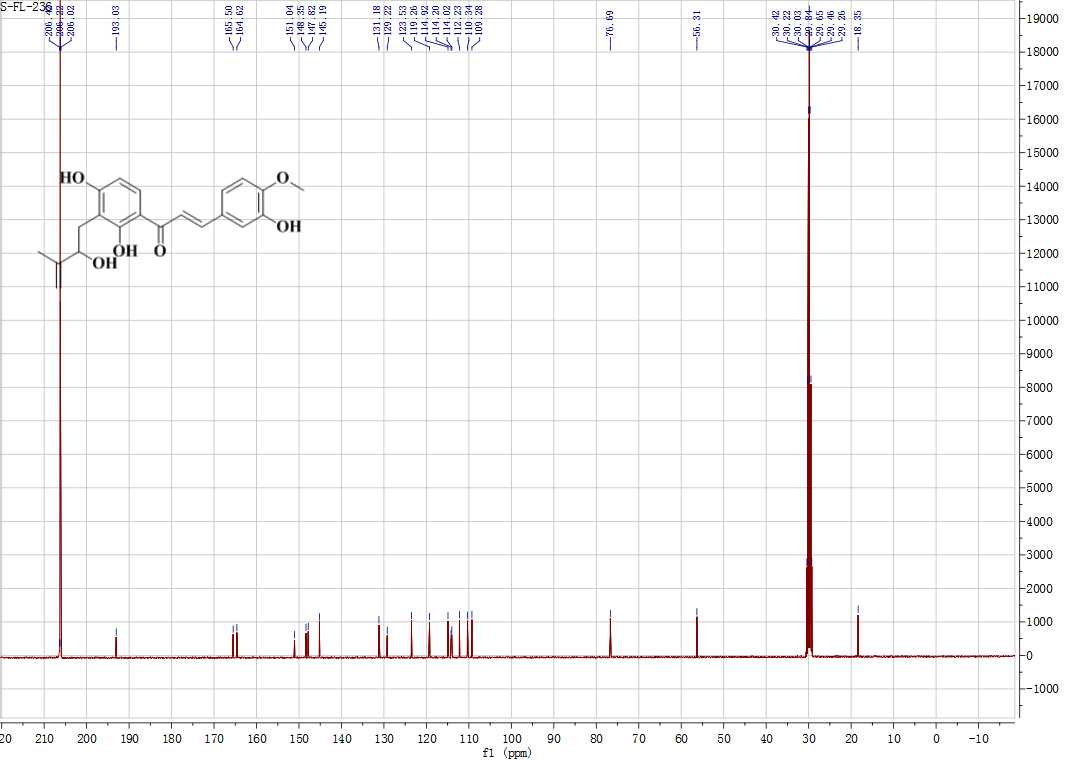


^13^C NMR of **4c**


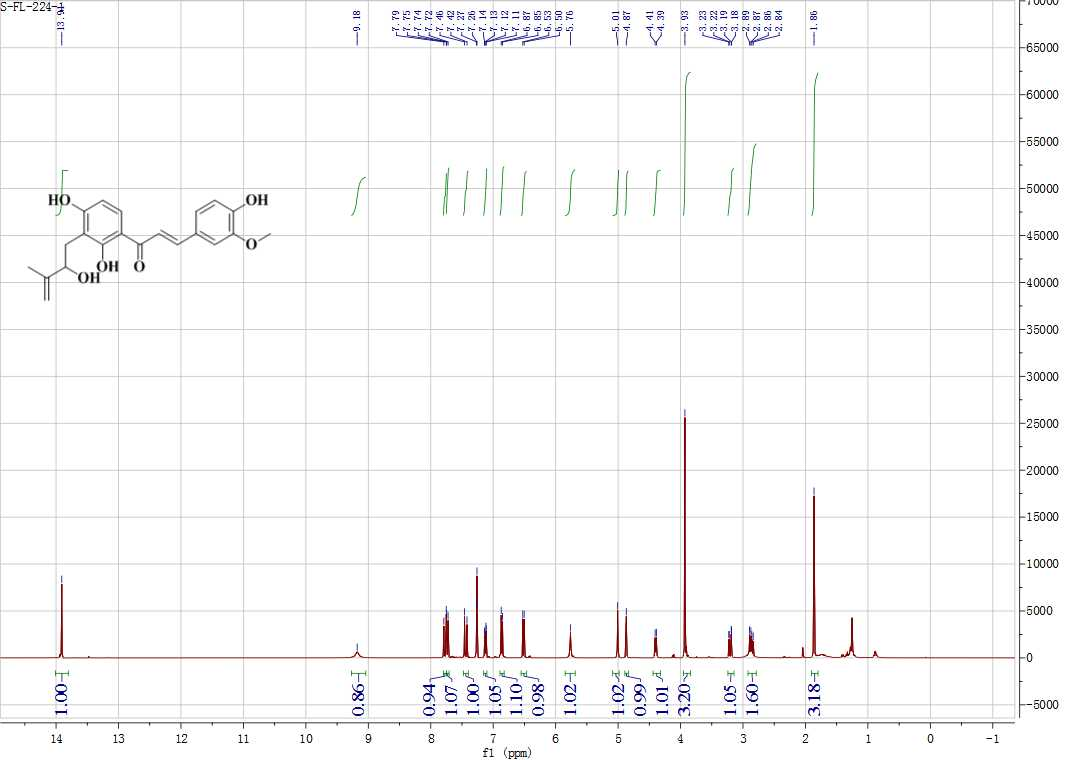


^1^H NMR of **4d**


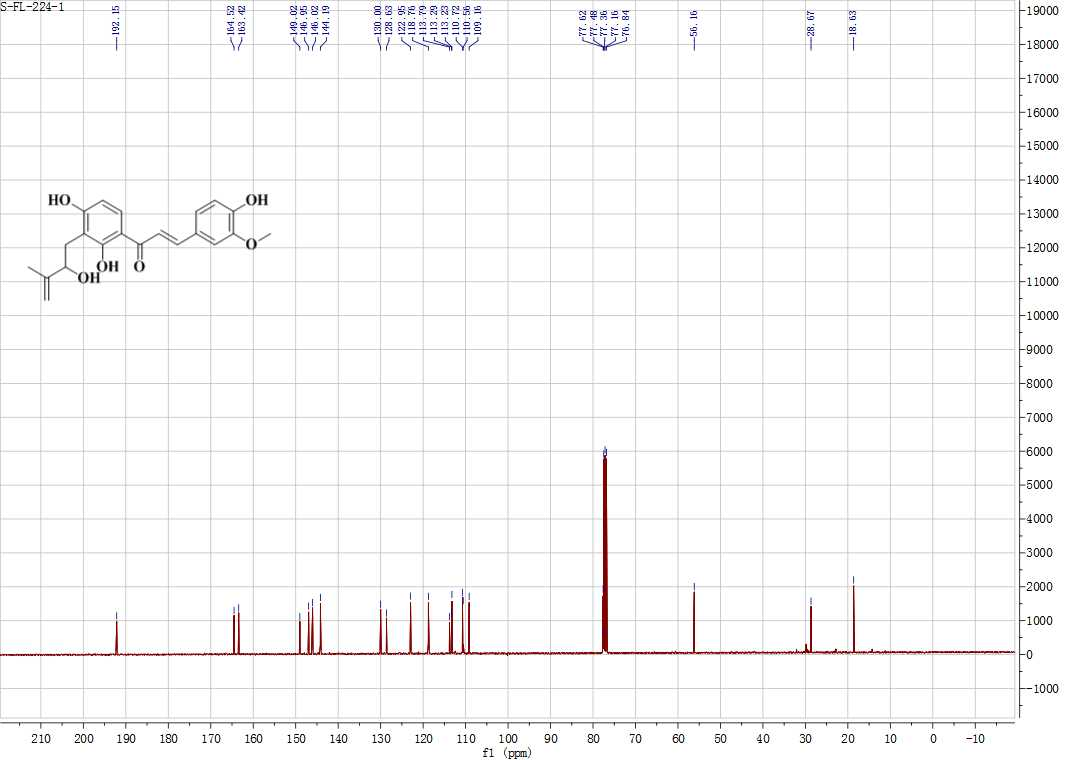


^13^C NMR of **4d**


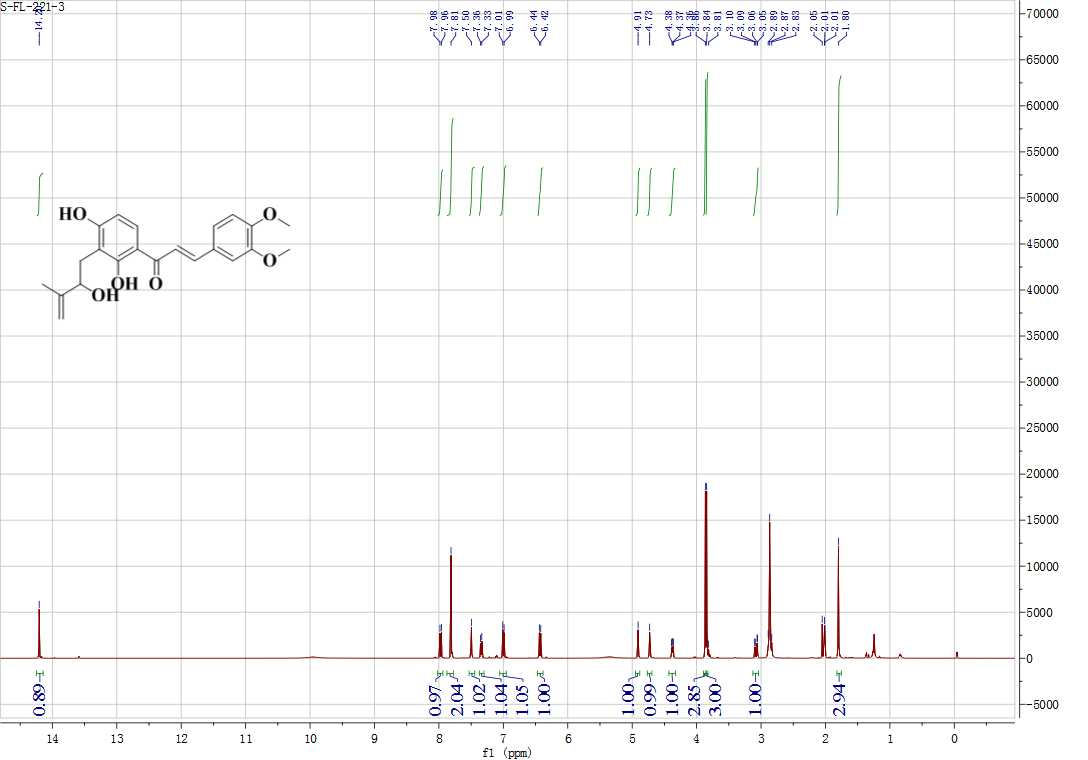


^1^H NMR of **4e**


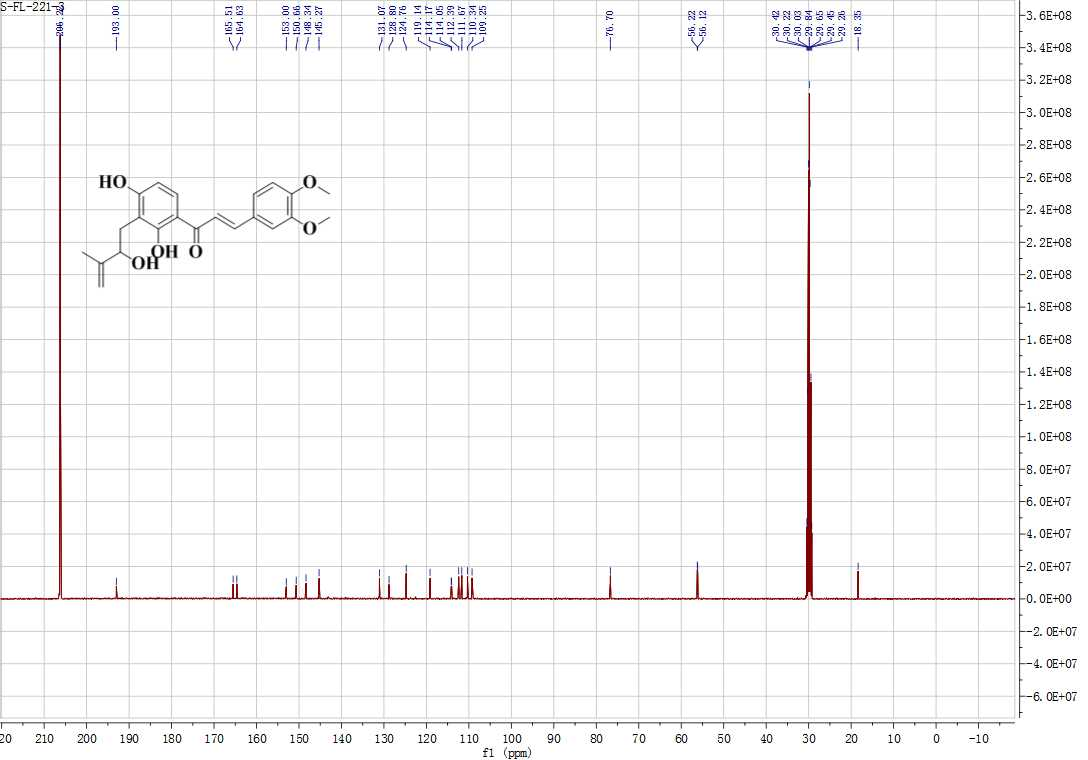


^13^C NMR of **4e**


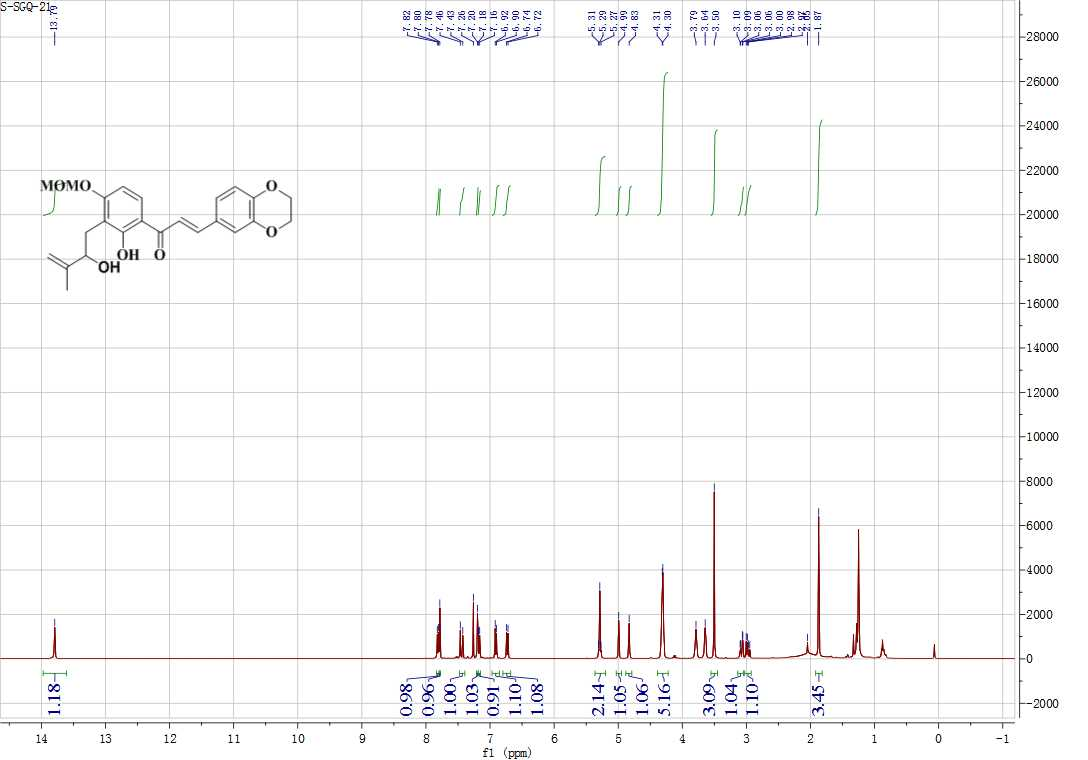


^1^H NMR of **3f**


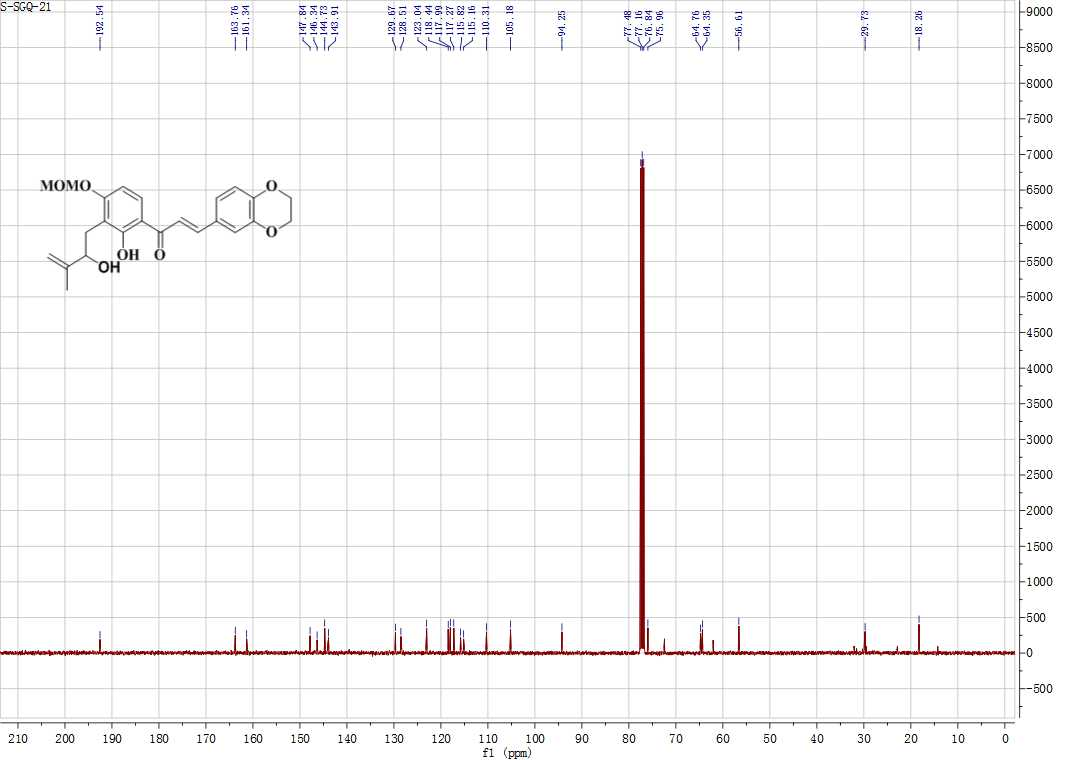


^13^C NMR of **3f**


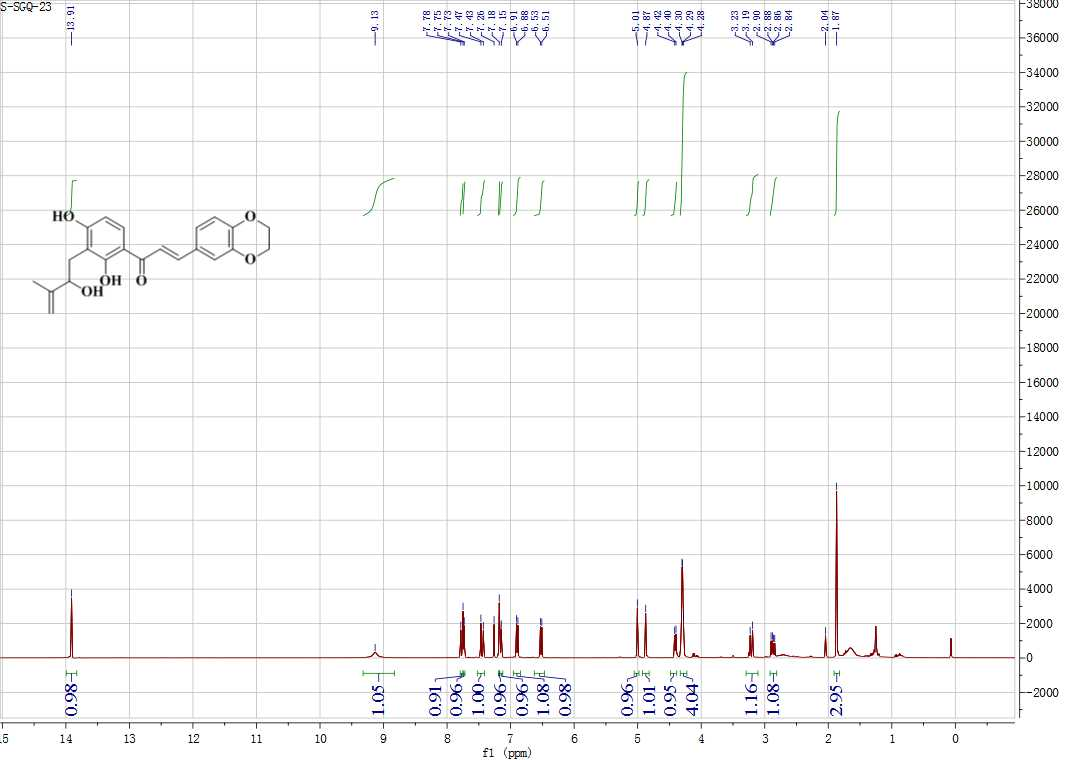


^1^H NMR of **4f**


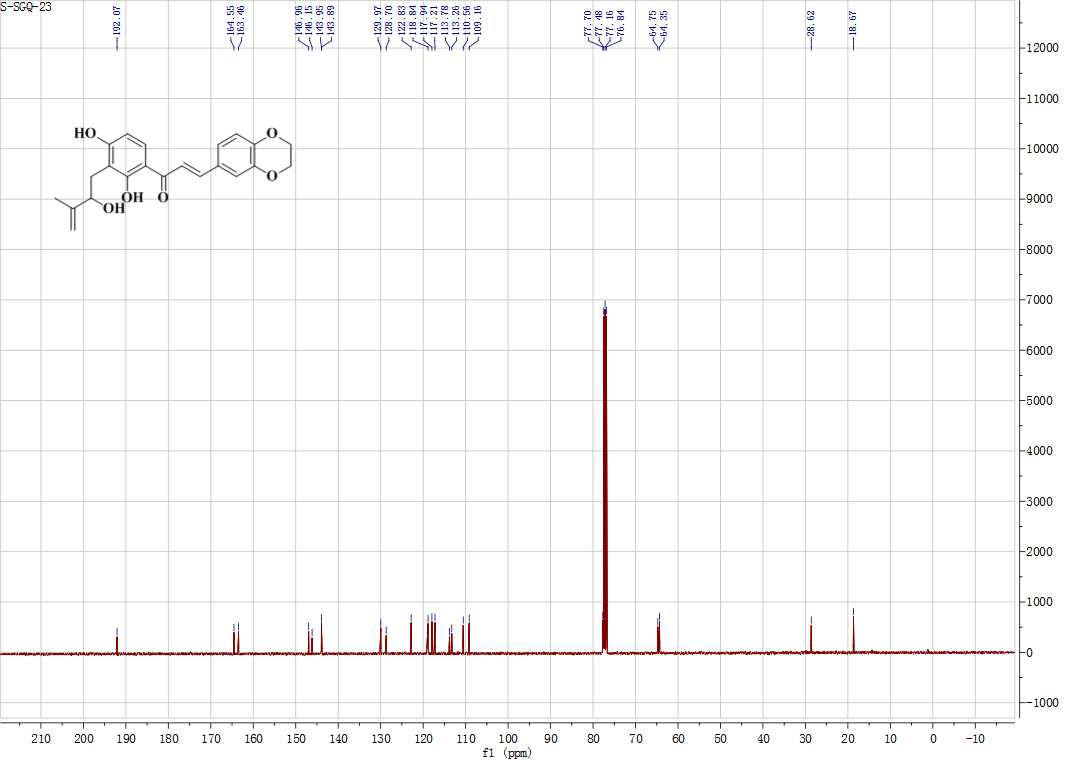


^13^C NMR of **4f**


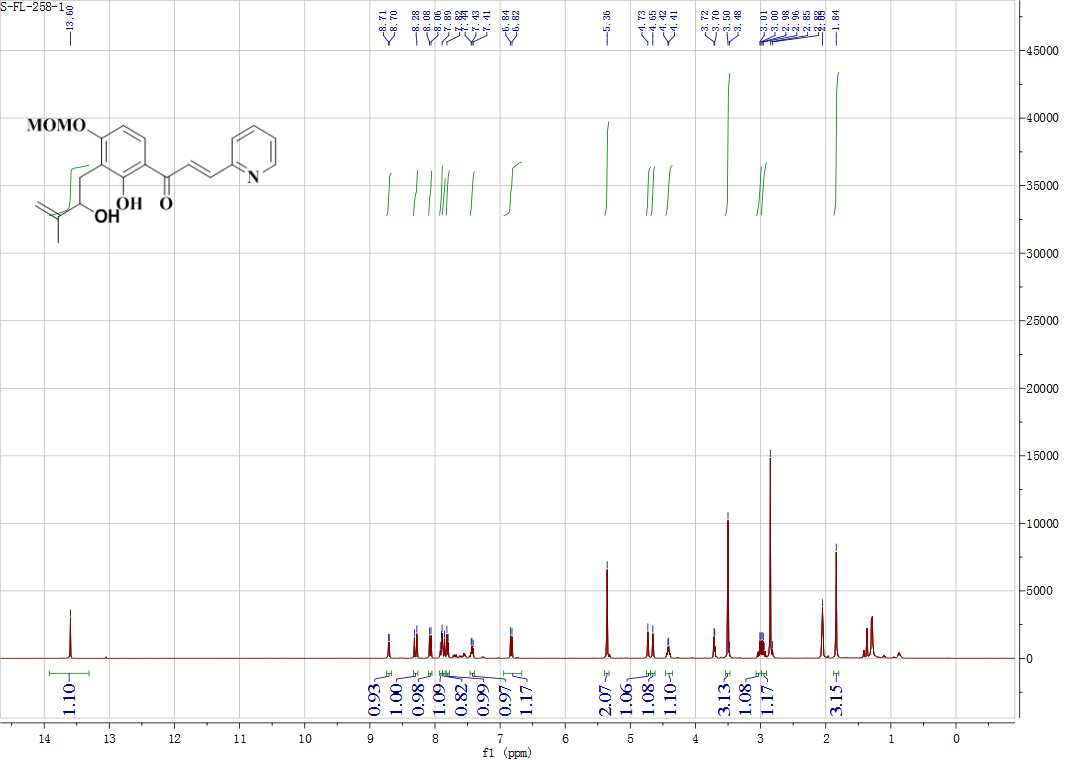


^1^H NMR of **3g**


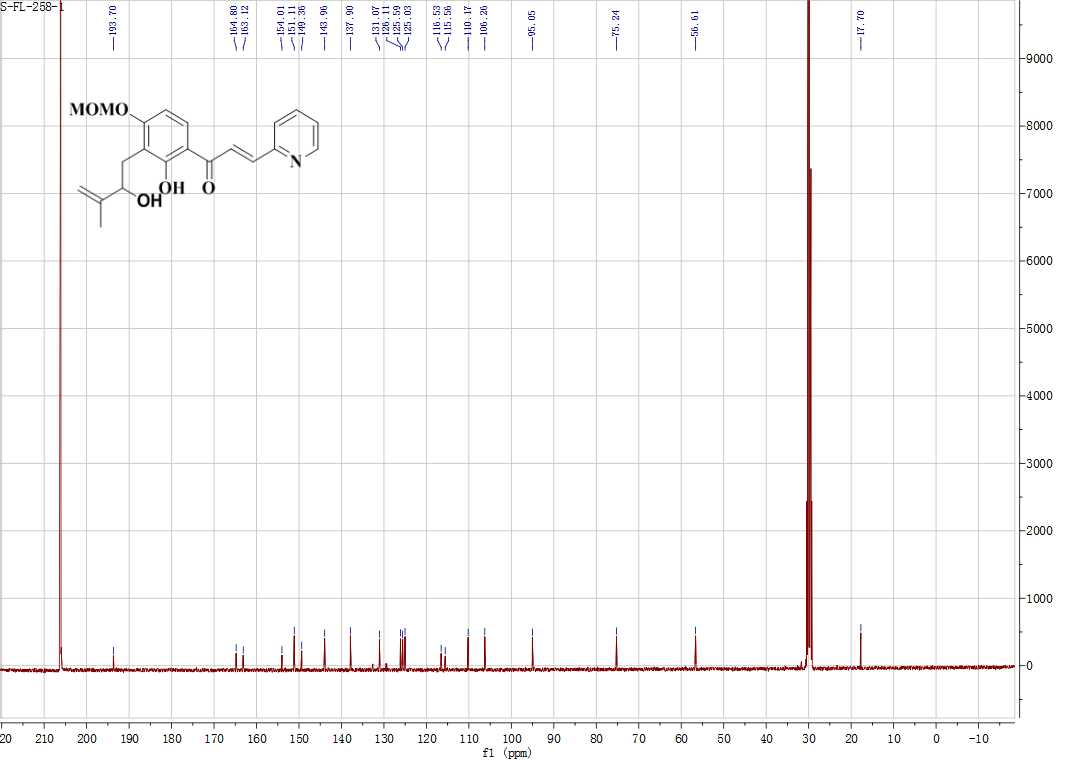


^13^C NMR of **3g**


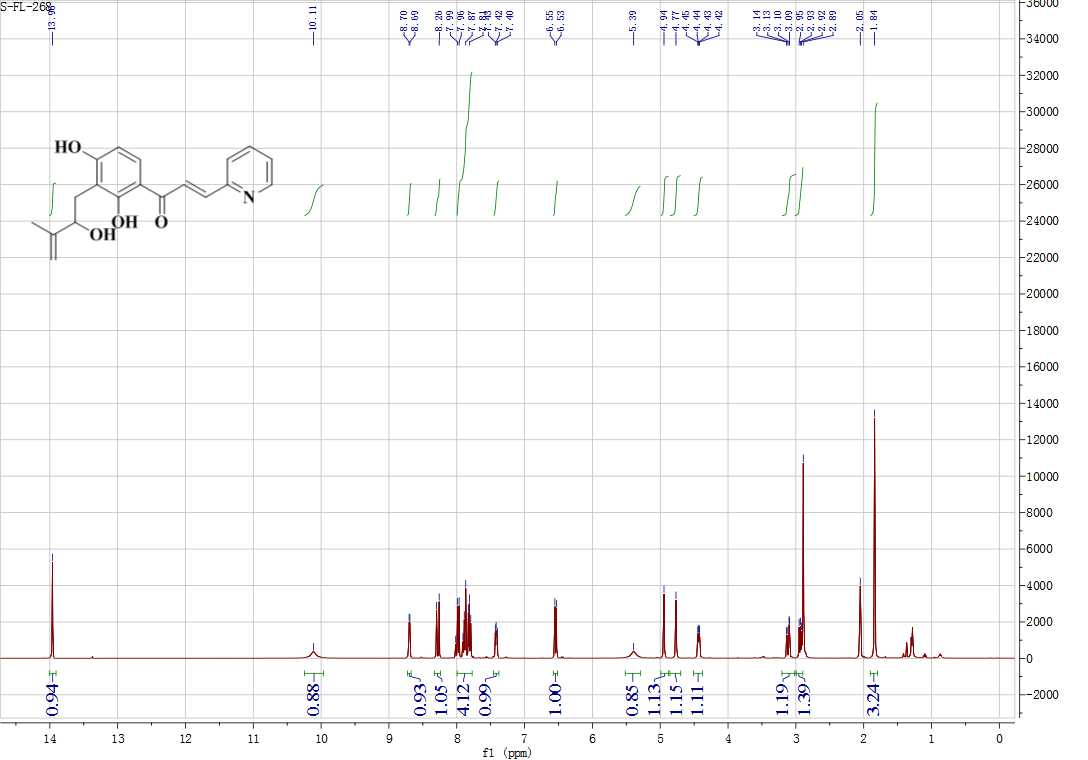


^1^H NMR of **4g**


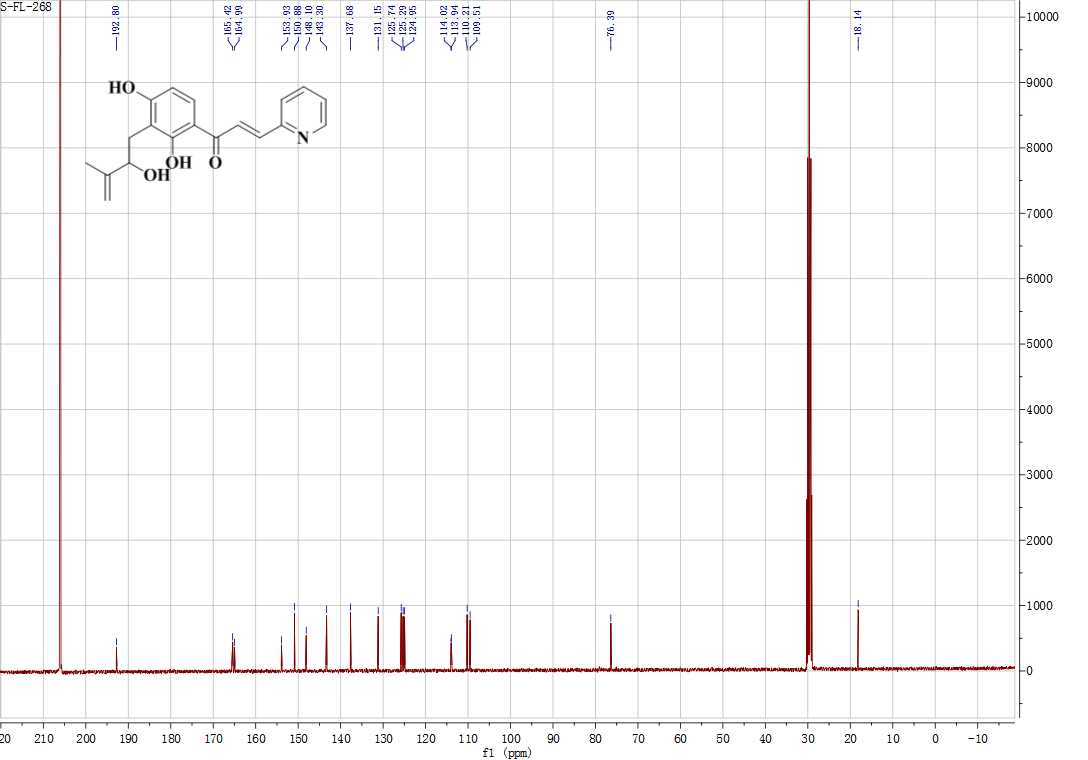


^13^C NMR of **4g**


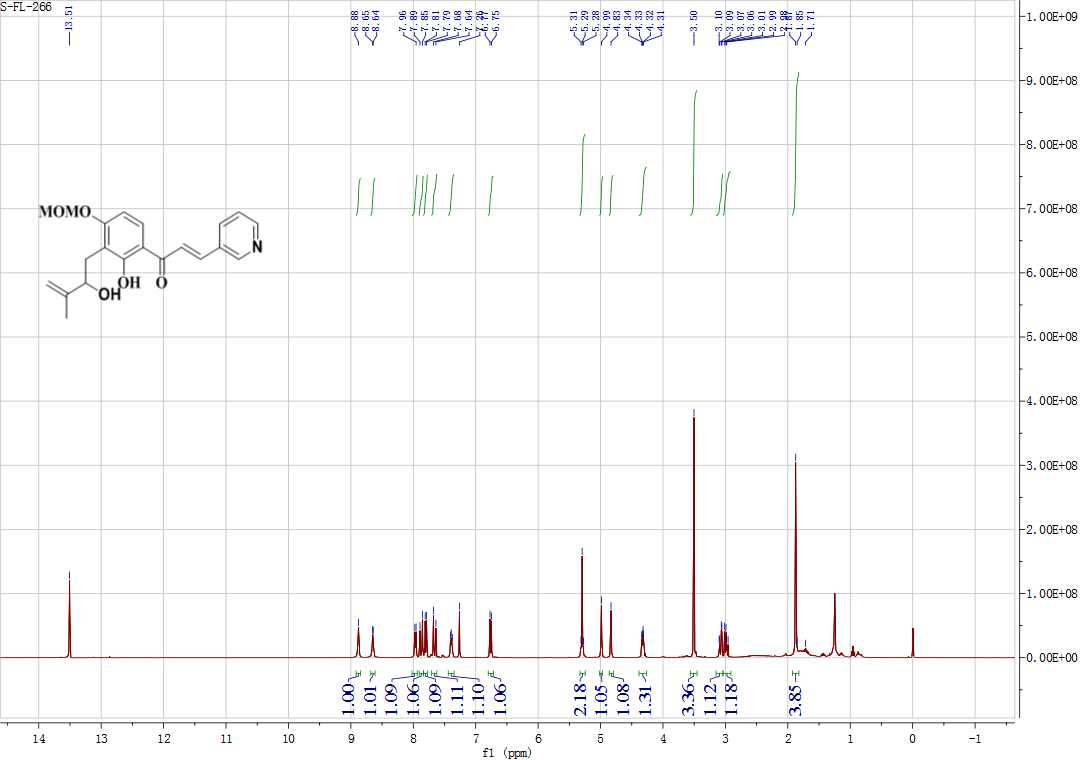


^1^H NMR of **3h**


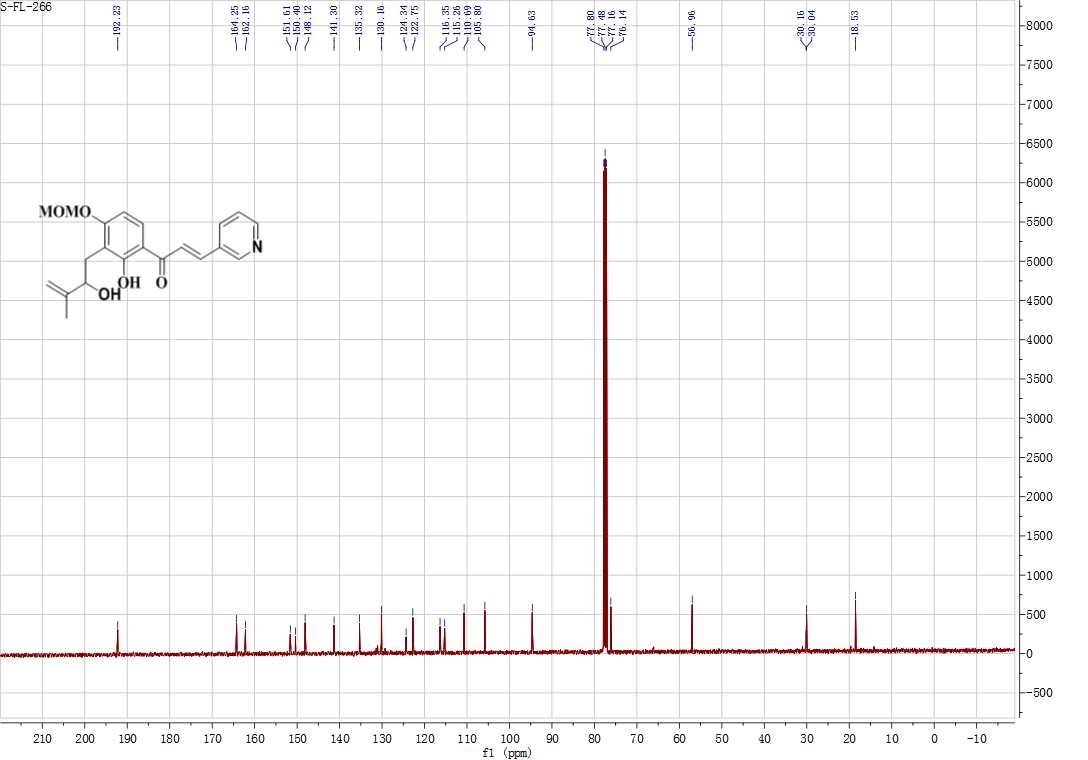


^13^C NMR of **3h**


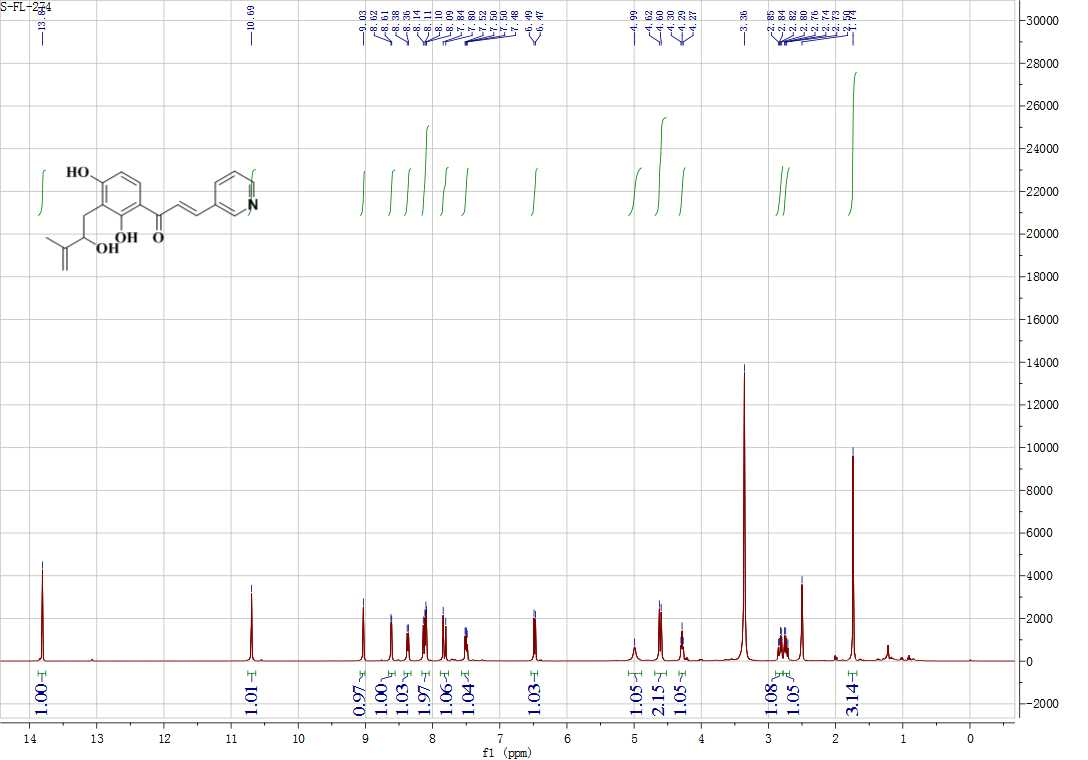


^1^H NMR of **4h**


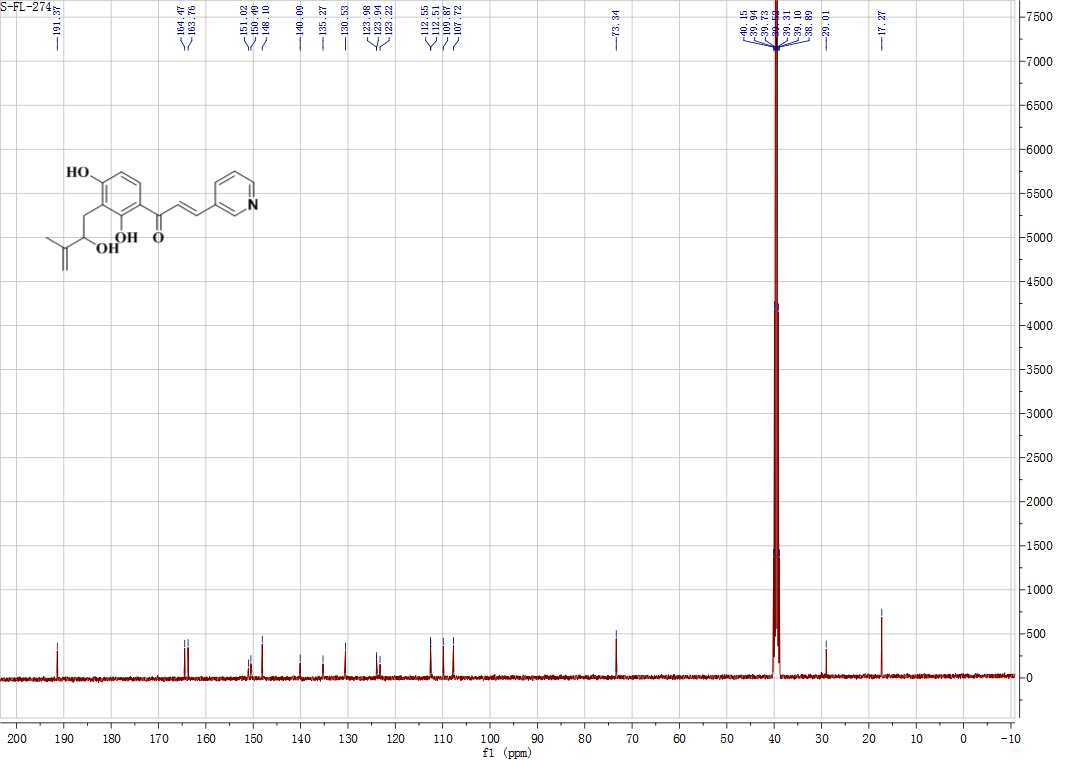


^13^C NMR of **4h**


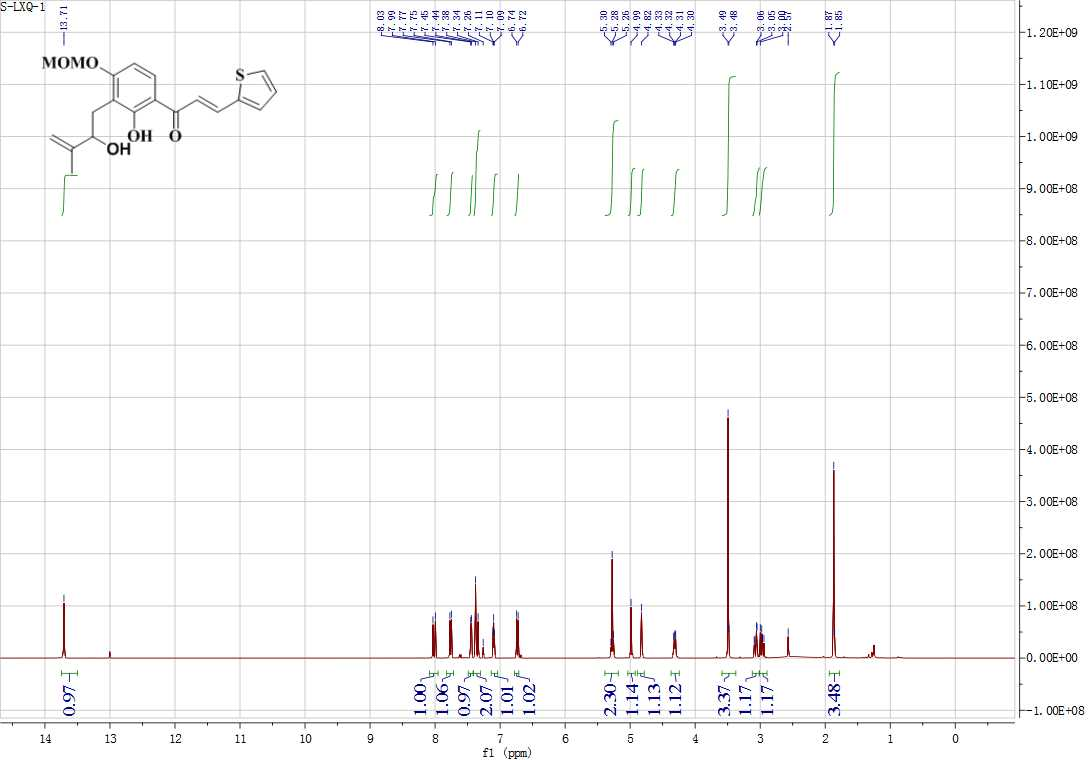


^1^H NMR of **3i**


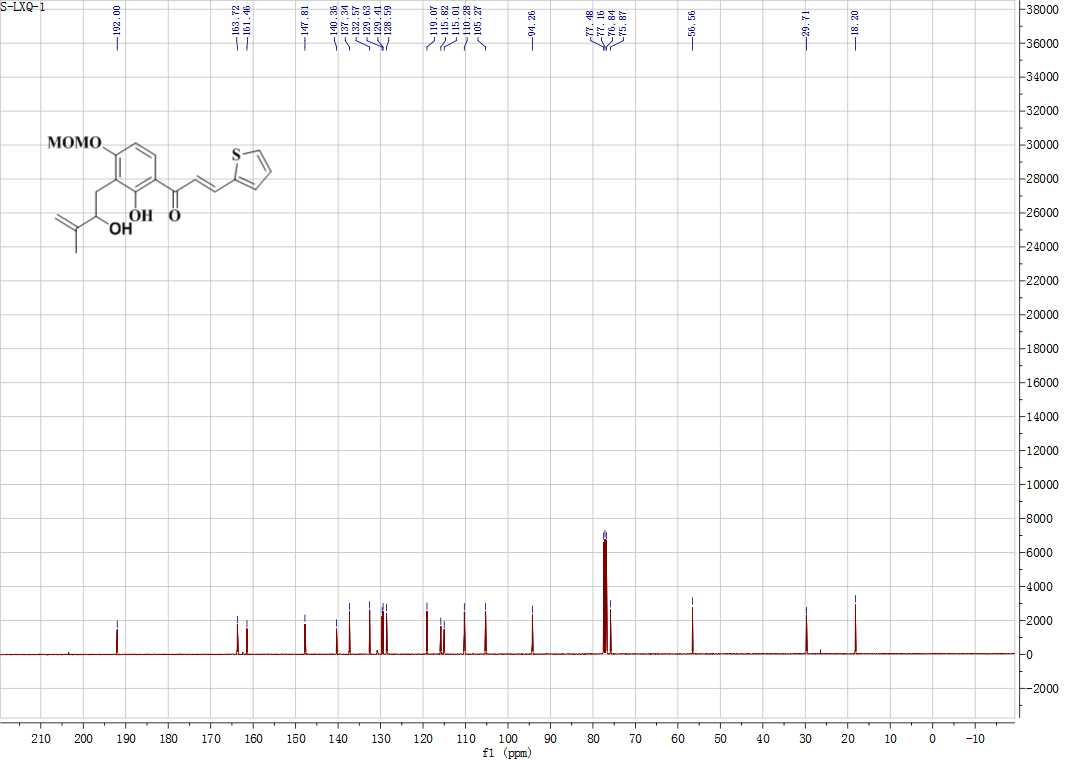


^13^C NMR of **3i**


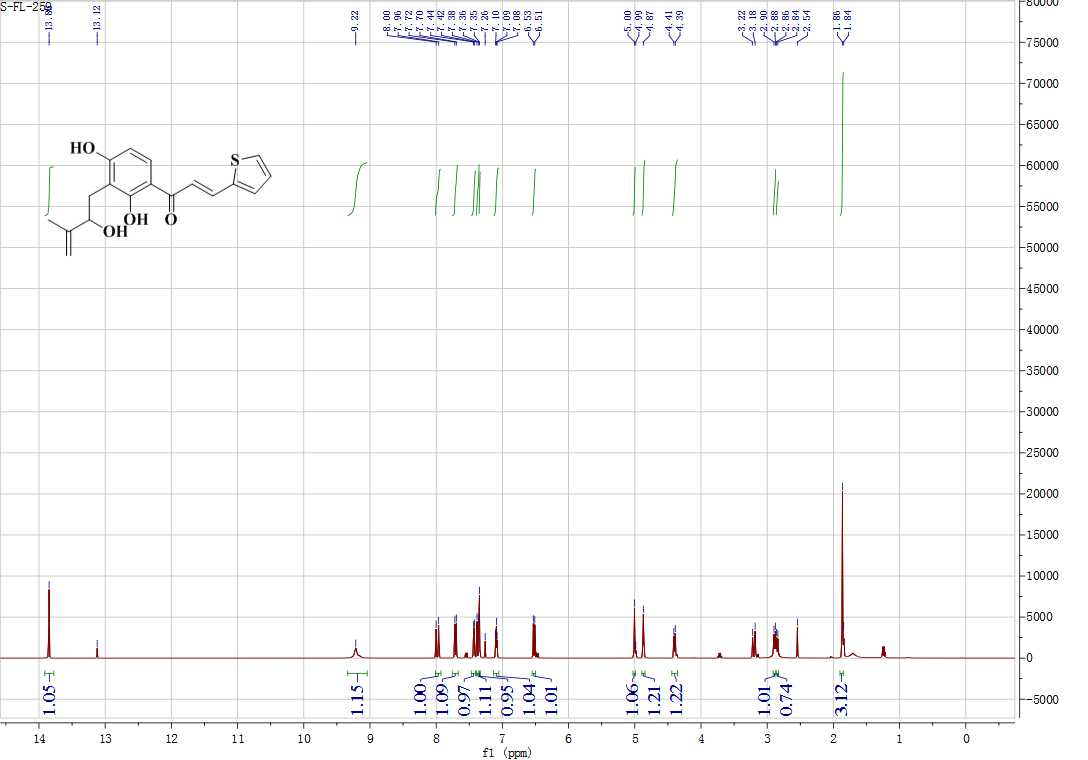


^1^H NMR of **4i**


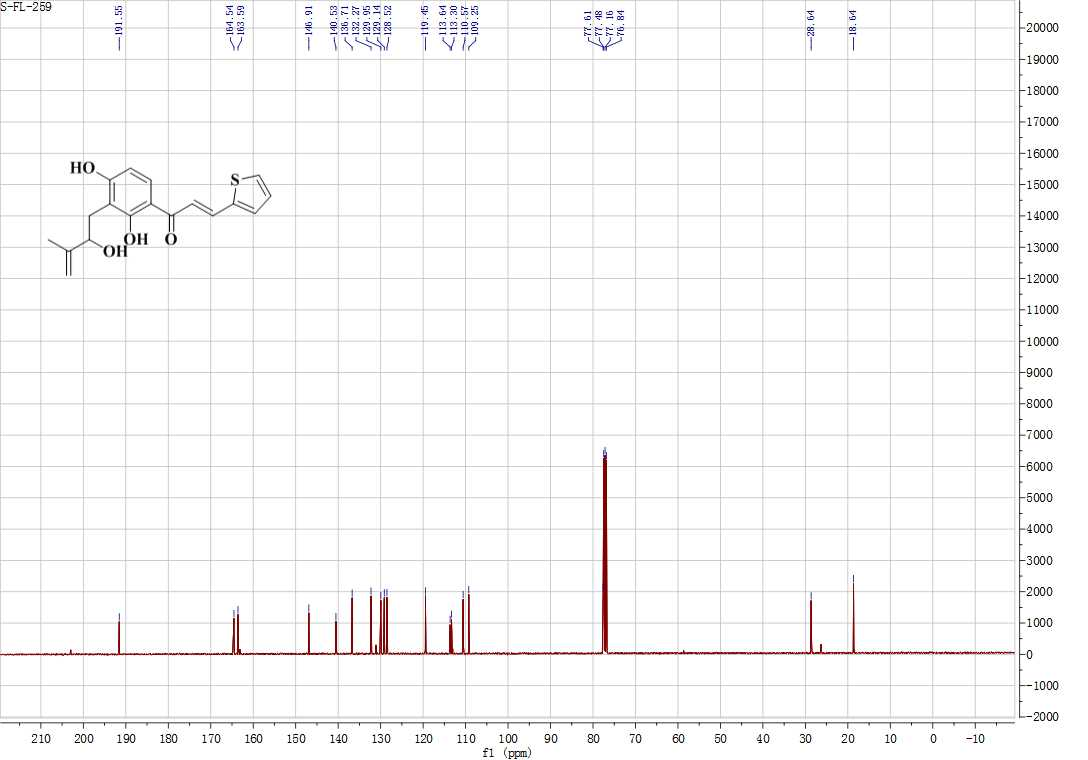


^13^C NMR of **4i**


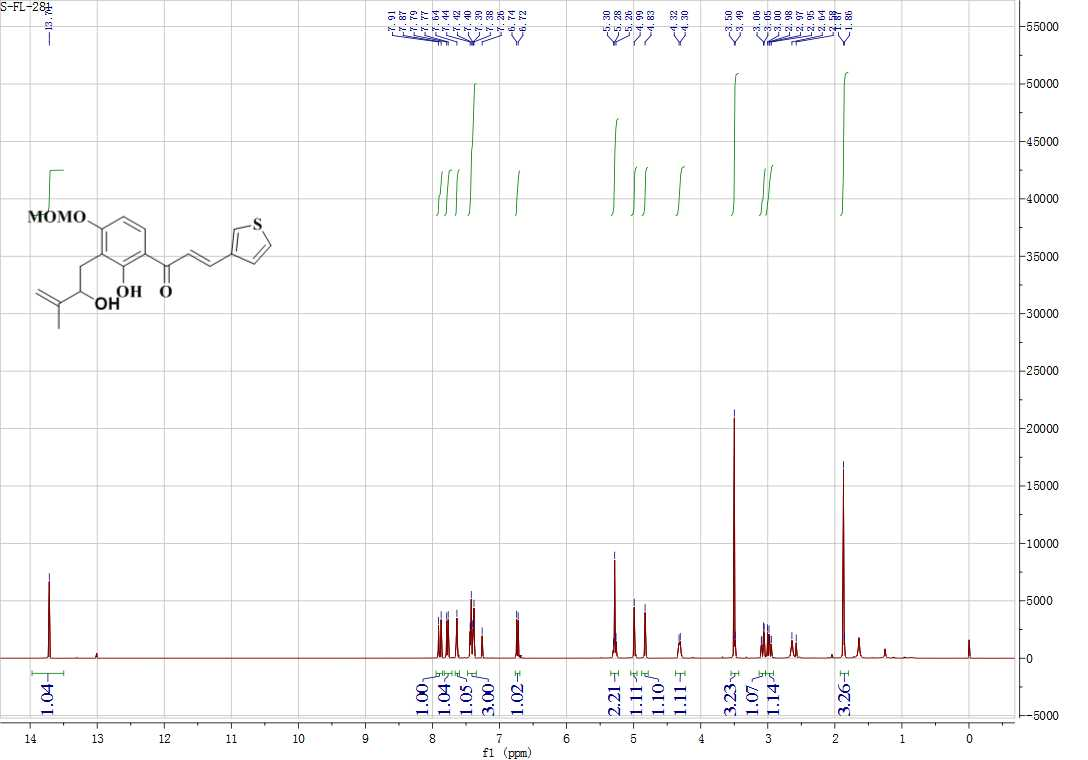


^1^H NMR of **3j**


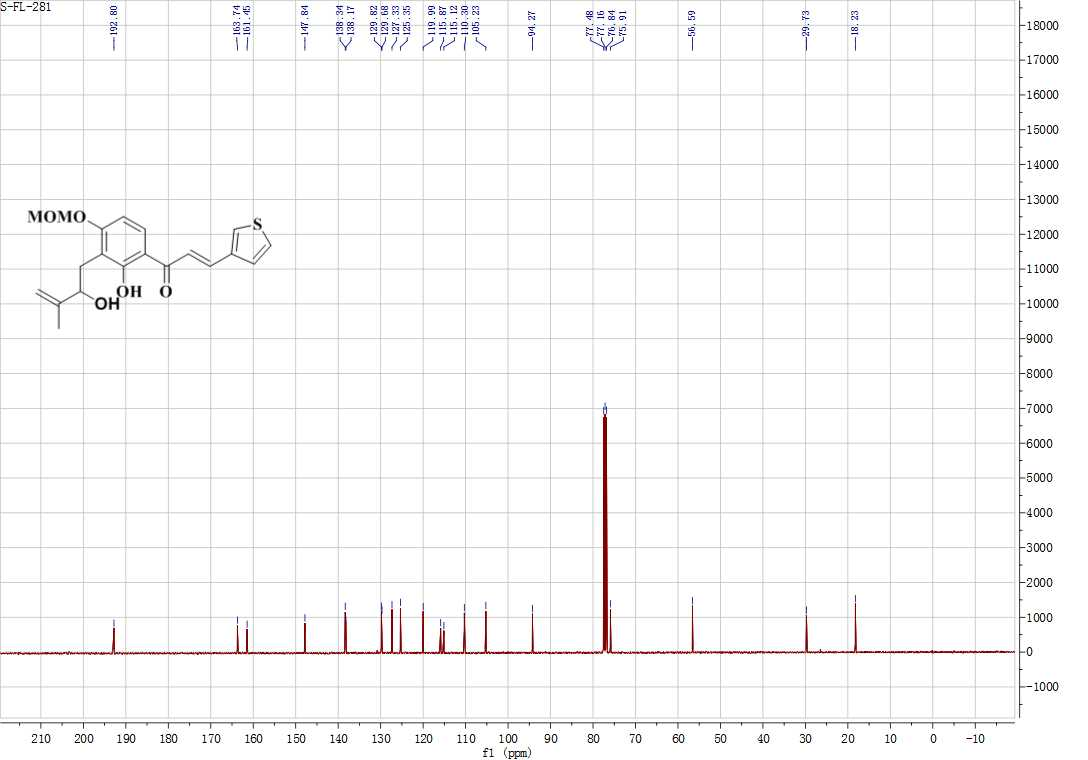


^13^C NMR of **3j**


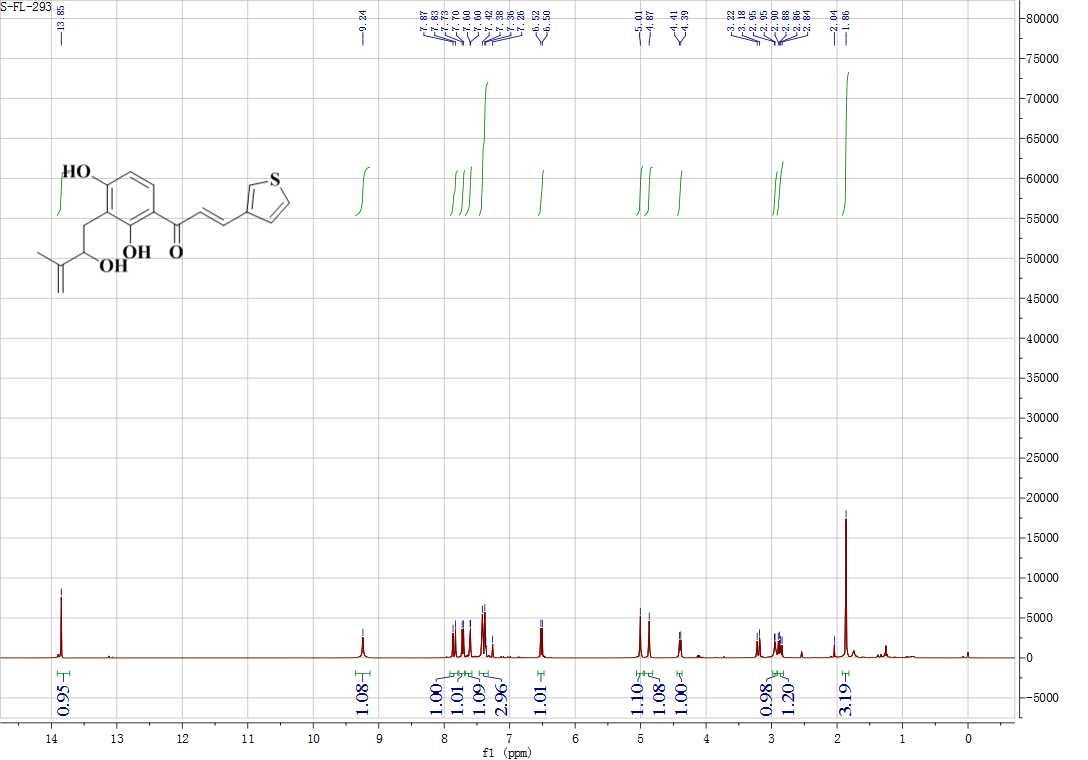


^1^H NMR of **4j**


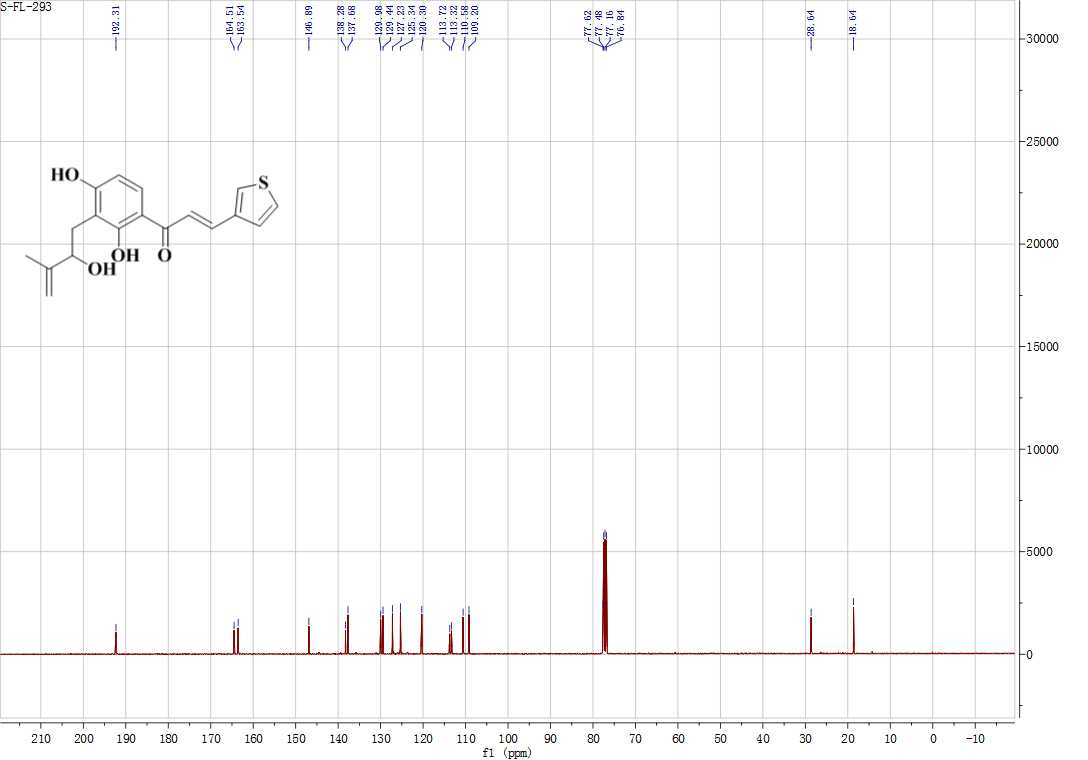


^13^C NMR of **4j**


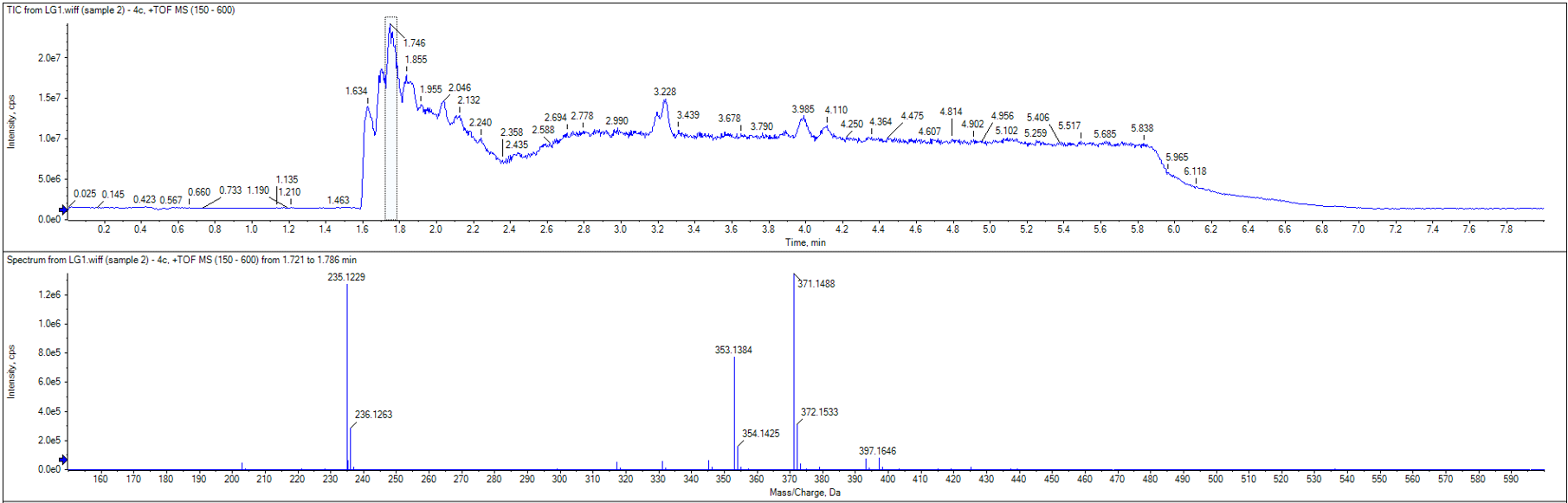


HRMS of **4c**


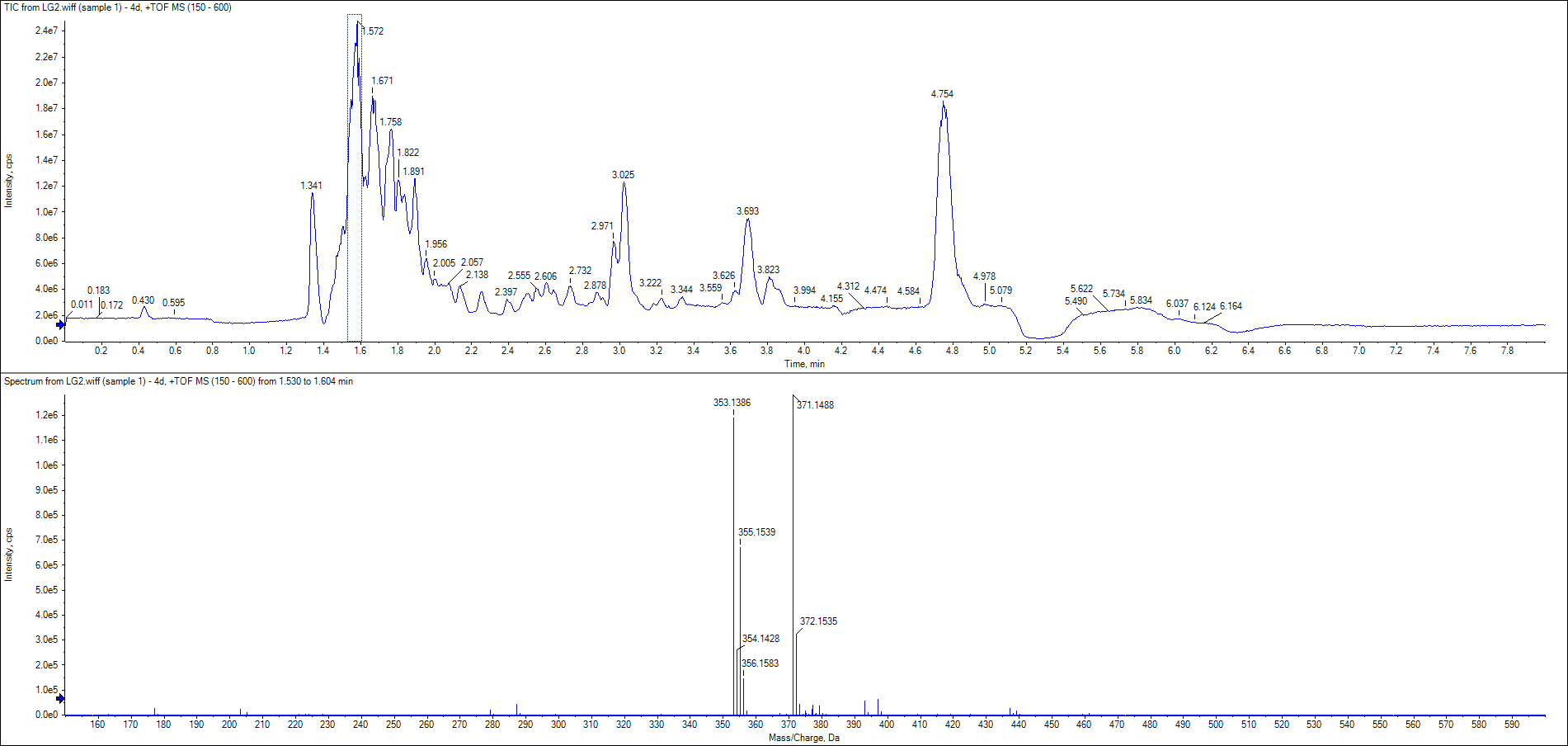


HRMS of **4d**
